# Osmoregulatory strategies of estuarine fish S*catophagus argus* in response to environmental salinity changes

**DOI:** 10.1186/s12864-022-08784-2

**Published:** 2022-07-30

**Authors:** Maoliang Su, Nanxi Liu, Zhengqi Zhang, Junbin Zhang

**Affiliations:** Shenzhen Key Laboratory of Marine Bioresource & Eco-Environmental Science, College of Life Sciences and Oceanography, Shenzhen University, Shenzhen, 518060 China

**Keywords:** Gill, iTRAQ, Environmental salinity stress, Osmoregulation, *Scatophagus argus*

## Abstract

**Background:**

*Scatophagus argus*, an estuarine inhabitant, can rapidly adapt to different salinity environments. However, the knowledge of the molecular mechanisms underlying its strong salinity tolerance remains unclear. The gill, as the main osmoregulatory organ, plays a vital role in the salinity adaptation of the fish, and thus relative studies are constructive to reveal unique osmoregulatory mechanisms in *S*. *argus*.

**Results:**

In the present study, iTRAQ coupled with nanoLC-MS/MS techniques were employed to explore branchial osmoregulatory mechanisms in *S. argus* acclimated to different salinities. Among 1,604 identified proteins, 796 differentially expressed proteins (DEPs) were detected. To further assess osmoregulatory strategies in the gills under different salinities, DEPs related to osmoregulatory (22), non-directional (18), hypo- (52), and hypersaline (40) stress responses were selected. Functional annotation analysis of these selected DEPs indicated that the cellular ion regulation (e.g. Na^+^-K^+^-ATPase [NKA] and Na^+^-K^+^-2Cl^−^ cotransporter 1 [NKCC1]) and ATP synthesis were deeply involved in the osmoregulatory process. As an osmoregulatory protein, NKCC1 expression was inhibited under hyposaline stress but showed the opposite trend in hypersaline conditions. The expression levels of NKA α1 and β1 were only increased under hypersaline challenge. However, hyposaline treatments could enhance branchial NKA activity, which was inhibited under hypersaline environments, and correspondingly, reduced ATP content was observed in gill tissues exposed to hyposaline conditions, while its contents were increased in hypersaline groups.* In vitro* experiments indicated that Na^+^, K^+^, and Cl^−^ ions were pumped out of branchial cells under hypoosmotic stress, whereas they were absorbed into cells under hyperosmotic conditions. Based on our results, we speculated that NKCC1-mediated Na^+^ influx was inhibited, and proper Na^+^ efflux was maintained by improving NKA activity under hyposaline stress, promoting the rapid adaptation of branchial cells to the hyposaline condition. Meanwhile, branchial cells prevented excessive loss of ions by increasing NKA internalization and reducing ATP synthesis. In contrast, excess ions in cells exposed to the hyperosmotic medium were excreted with sufficient energy supply, and reduced NKA activity and enhanced NKCC1-mediated Na^+^ influx were considered a compensatory regulation.

**Conclusions:**

*S. argus* exhibited divergent osmoregulatory strategies in the gills when encountering hypoosmotic and hyperosmotic stresses, facilitating effective adaptabilities to a wide range of environmental salinity fluctuation.

**Supplementary Information:**

The online version contains supplementary material available at 10.1186/s12864-022-08784-2.

## Introduction

Estuaries are characterized by wide fluctuations in salinity due to the mixture of fluvial and marine waters, and estuarine organisms have to cope with variable salinities, triggering corresponding changes in growth, reproduction and other physiological processes [1, 2]. The fish *Scatophagus argus* is an estuarine resident notable for its high tolerance to environmental salinity change, and the culture of this species has spread rapidly throughout the coastal areas of South China in recent years [3–6]. Our previous study has revealed that *S. argus* develops specific strategies of osmotic and ion regulation to adapt to waters of varying salinity, and the fish is an ideal model to study physiological responses to environmental salinity stress [7]. Unfortunately, the molecular mechanisms of its high salinity tolerance are poorly understood so far. Thus, relative studies are constructive to reveal unique osmoregulatory mechanisms in *S*. *argus*, and ultimately for promoting its commercial aquaculture.

Proteins serve crucial functions essentially in all biological processes. Alterations in the proteome directly affect the physiological status of fish, and physiological processes are related to a complex network of changes at the protein level [8–13]. However, the lack of specific antibodies has been the major drawback for physiological studies in non-model fish. Comparative proteomics study provides an alternative approach to detect proteome changes in physiological responses to environmental stresses in some non-model animals, such as *Oreochromis mossambicus*, *Ctenopharyngodon idellus* and *Gasterosteus aculeatus* [14–16]. Isobaric tags for relative and absolute quantitation (iTRAQ) combined with liquid chromatography-tandem mass spectrometry (LC–MS/MS) have become popular proteomics technology for the direct quantitation and comparison of protein expression levels with great efficiency and accuracy [17]. The identification of differentially expressed proteins (DEP) through proteomic analysis can yield a global, integrated view of physiological processes in fish undergoing diverse environmental conditions [16, 18, 19].

High salinity adaptation is primarily achieved *via* branchial osmoregulation, which is a common trait found in estuarine residents [20]. The fish gill is a multipurpose organ that directly contacts the external milieu and is considered the major tissue for osmoregulation during rapid shifts to hypo- or hypersaline conditions [15, 21, 22]. In the osmoregulatory process, fish gills are critical to maintain the ionic compositions of body fluids encountering with salinity fluctuations in aquatic environments [15]. Fish in hypersaline water passively lose water and gain salt and need to replenish water by drinking and actively excreting the majority of monovalent ions back to the environment, whereas in hyposaline water they actively ingest ions across branchial epithelia to compensate for monovalent ion loss, primarily sodium ion (Na^+^) loss [21–24]. Ionocytes (also called mitochondrion-rich cells [MRCs]) in branchial epithelia are majorly responsible for adenosine triphosphate- (ATP-) dependent active ion transport [22, 25]. This regulatory mechanism depends on a lot of ion channels, pumps and exchangers on the membranes of branchial MRCs [26, 27]. Na^+^-K^+^-ATPase (NKA) and Na^+^-K^+^-2Cl^−^ cotransporter (NKCC), explicitly localized to branchial MRCs, are responsible for maintaining ionic balance and are generally considered as sensitive molecular biomarkers to understand the osmotic stress status of euryhaline fish [28–30]. NKA drives various ion-transporting processes, establishing electrochemical gradients across the cell membrane, which is a major source of energy consumption in cells through the hydrolysis of ATP [22, 31, 32]. The basolateral located NKA pumps three Na^+^ ions outward in exchange for two K^+^ ions in every pump cycle, creating low intracellular Na^+^ and a highly negative charge within the cell [22, 33]. NKCC cooperates with the NKA to actively reabsorb ions into branchial cells either from the blood or environment based on salinity [30, 34–36]. In the gills of euryhaline fish, the expression levels of NKAα1 and NKCC1 are generally supposed to be up-regulated with increased salinity and decreased when salinity dropped [28, 37]. However, conflicting opinions have been reported in some species [32]. The protein abundance of NKAα1 and NKCC1 increase under hypersaline conditions and remain stable undergoing hyposaline stress in *Poecilia latipinna* and *Lateolabrax maculatus* [32, 38]. Different ion regulatory mechanisms may be potentially responsible for differences in environmental salinity tolerance in fish, and thus further studies are needed to be undertaken to explore branchial osmoregulatory functions.

In this study, iTRAQ coupled with nanoLC-MS/MS was used for relative quantitation of the protein abundance in gill tissue from different salinity acclimated *S. argus*, with the aim to obtain protein profiles related to its high salinity tolerance, and relative osmoregulatory functions were verified by *in vivo* and *in vitro* experiments. In fish, previous studies about osmoregulatory functions are mainly performed based on *in vivo* experiments in the gill tissues, and thus molecular mechanisms of branchial osmoregulation are still unclear so far [28, 39]. Primary cells, isolated directly from the animal tissue, are commonly considered more biologically relevant than cell lines as the biological response may be closer to the *in viv*o situation [40]. Herein, for further exploring the osmoregulatory strategies of gills under salinity stress, the primary culture of branchial cells *in vitro* was performed. The main goals of this study were as follows: (1) to utilize a mass spectrometry-based iTRAQ labeling approach to analyze the branchial proteomics profiles of *S. argus* after exposure to different salinities, (2) to identify candidate functional proteins related to osmoregulation, (3) to evaluate the relevance between candidate proteins and the salinity stress response, and (4) to analyze osmoregulatory strategies under diverse salinity environments using *in vivo* and *in vitro* experimental techniques.

## Methods

### Collection & treatment of fish and sampling procedure

**Fig. 1:**
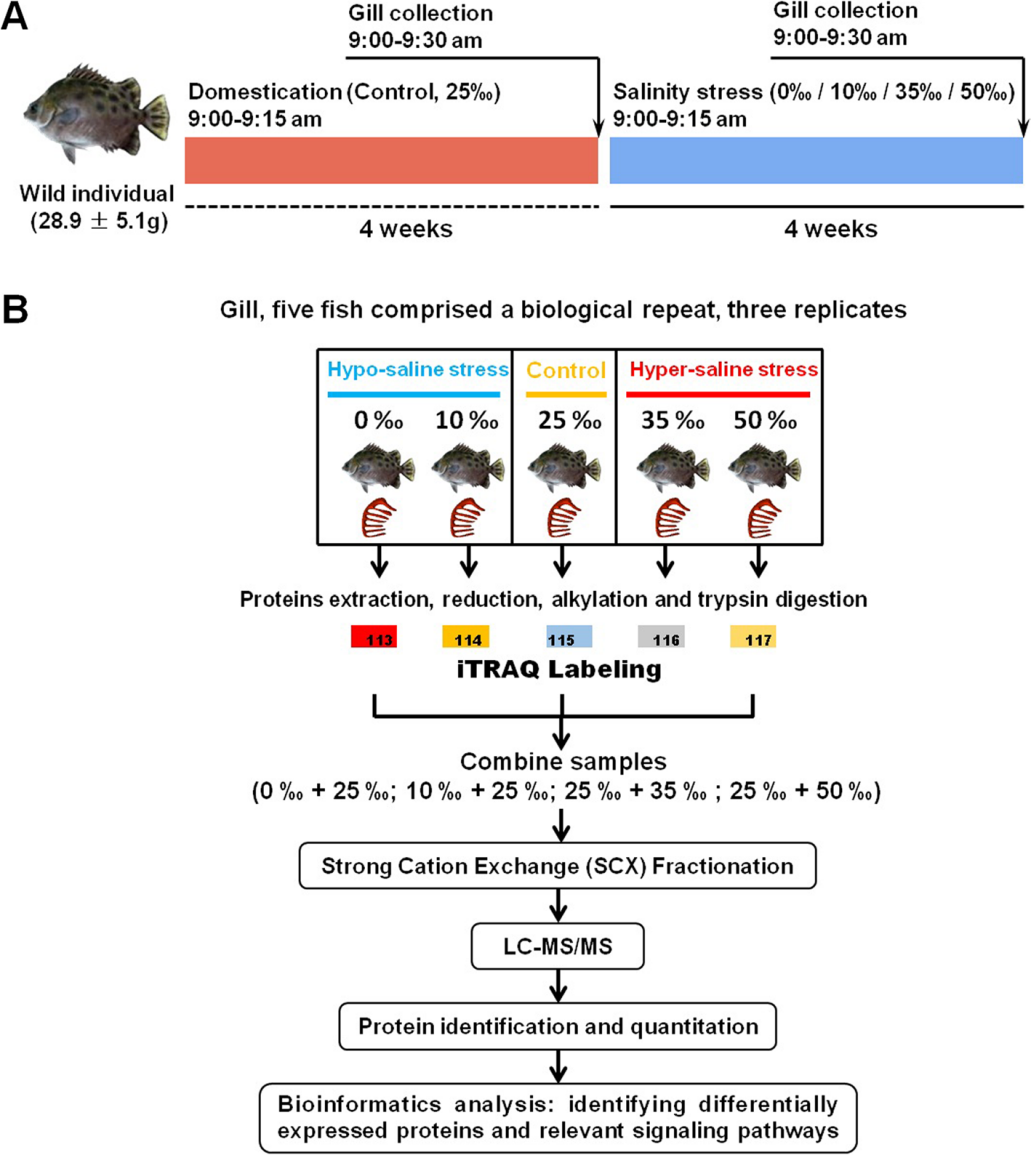
Experimental design and proteomic study of the gills of *Scatophagus argus* under long-term salinity stress. **A** Treatment and sampling; **B** workflow of the proteomic study. Three sets of biological replicates were analyzed by iTRAQ-coupled nanoLC-MS/MS to examine the proteomic changes in gill tissues of *S. argus* in response to environmental salinity stress (0‰ and 10‰ as the hyposaline stress groups, 25‰ as the control group, and 35‰ and 50‰ as the hypersaline stress groups)

Individuals of *S. argus* (34.8 ± 6.7 g) were collected from the sea (~ 25‰ salinity) near Zhuhai, Guangdong Province, China. Fish were reared in the tank (1.0 m × 1.0 m × 1.0 m) containing 25‰ seawater (SW) for 4 weeks. The water temperature was maintained at 28 ± 1 °C. The fish were fed with live brine shrimp (*Artemia nauplii*) twice a day. Feeding was stopped 24 h before sampling to minimize the influence of food on the blood test results. For long-term salinity stress experiments, these individuals were assigned to 5 groups: 2 hyposaline groups (0‰ freshwater [FW] and 10‰ SW, *n* = 15), 2 hypersaline groups (35‰ SW and 50‰ SW, *n* = 15), and a control group (25‰ SW, *n* = 15). Fish in the hypo- and hypersaline groups were transferred from 25‰ SW to new tanks, and the salinity was adjusted to the designed salinities by a gradual salinity increase or deduction of 2‰ salinity per day during two weeks. An additional acclimation of four weeks to new conditions was applied before stress experiments. The individuals in 25‰ SW were also transferred to a new tank with the same salinity for four weeks, which were served as the control group. No deaths occurred during the period of stress experiments. Before the experiment, fish were weighted (Fig. S1), and then anesthetized with 50 mg/L 3-Aminobenzoic acid ethyl ester methanesulfonate (MS-222, Sigma-Aldrich, USA) and subsequently euthanized by a sharp blow to the head. Fifteen fish in each group were divided into three sets, each of which (5 fish) represented one biological replicate. Fresh gill tissues from each set were sampled (Fig. 1A), and frozen immediately in liquid nitrogen. Animal welfare and experimental procedures were performed in accordance with the Guide for the Care and Use of Laboratory Animals (Ministry of Science and Technology of China, 2006) and were approved by the Animal Ethics Committee of Shenzhen University (Reference No. 2014–162). Meanwhile, our study was carried out in compliance with the ARRIVE guidelines.

### Protein extraction and iTRAQ labeling

Approximately 0.1 g of each frozen gill tissue was ground to a fine powder in liquid nitrogen and homogenized in extraction buffer (4% SDS, 1 mM DTT, 150 mM Tris–HCl, pH = 8). The homogenates were sonicated and subsequently centrifuged at 16,000 × g for 10 min at 25 °C. The protein content was determined using a BCA protein assay kit (Pierce, USA).

iTRAQ labeling was conducted according to the published protocol [41]. An overview of the experimental design and workflow is shown in Fig. 1B. In brief, 100 μg of each sample was denatured, reduced, and alkylated as described in the iTRAQ protocol (Applied Biosystems, USA). Each sample was digested with 0.1 μg/μL trypsin at 37 °C overnight, and the digested peptides were dried by vacuum centrifugation. Peptides of the 5 groups (0‰, 10‰, 25‰, 35‰, and 50‰) were labeled with iTRAQ tags 113, 114, 115, 116, and 117 respectively, according to the manufacturer’s instructions (Applied Biosystems, USA). Subsequently, these peptides of each group were pooled and vacuum-dried for the following experimental procedure.

### Peptide fractionation and LC–MS/MS analysis

Strong cation exchange (SCX) fractionation of the iTRAQ-labeled peptide mixture was carried out by SCX chromatography [42] using the AKTA™ Purifier system (GE Healthcare, CHN) as described in a previously published protocol [41]. Thirteen fractions were obtained finally, and then desalted, dried, and stored at -80 °C.

LC–MS/MS analysis was performed on a Q Exactive mass spectrometer coupled to an Easy nLC (Thermo Finnigan, USA) [42, 43]. For each nanoLC-MS/MS analysis, a 10 μL peptide sample was injected. The peptide mixture (5 μg) was loaded onto a C18-reversed-phase column (Thermo Scientific Easy Column, 10 cm × 75 μm, 3 μm; Thermo Finnigan, USA) in buffer A (0.1% formic acid) and separated using a linear gradient of buffer B (80% acetonitrile and 0.1% formic acid) at a flow rate of 250 nL/min over 140 min, controlled by IntelliFlow technology [42]. Mass spectrometry (MS) data were acquired using a data-dependent top 10 method by dynamically choosing the most abundant precursor ions from the survey scan (300 − 1800 m*/z*) for high-energy collision-induced dissociation (HCD) in the C-trap and collision-induced dissociation (CID) in LTQ [44]. Determination of the target value was based on predictive automatic gain control (pAGC). The dynamic exclusion duration was 60 s. Survey scans were acquired with a resolution of 70,000 (at 200 m*/z*) in profile mode, with a maximum ion accumulation time of 2 s and a target value of 1e^6^. For HCD spectra, the resolution was set to 17,500 (at 200 m/z) in centroid mode, with a target value of 5e^4^, a normalized collision energy of 50%, and an activation time of 10 ms. For CID spectra, the normalized collision energy, activation Q, and activation time were set at 35%, 0.25, and 10 ms, respectively [42]. The instrument was run with peptide recognition mode enabled.

### Protein identification and quantitation

For peptide data analysis, raw mass data were processed using Proteome Discover 1.4 software and searched in the database of Eupercaria protein sequence database (20–02-2021, 693,004 entries) on the UniProt website (http://www.uniprot.org) and in the decoy database using Mascot 2.3.02 (Matrix Science, UK). For protein identification, the search parameters were set as follows: monoisotopic mass, peptide mass tolerance of 20 ppm, fragment mass tolerance of 0.1 Da, trypsin as the digestion enzyme with an allowance for a maximum of two missed cleavages, carbamidomethyl (C) and iTRAQ8plex modification (K and N-terminus) as a fixed modification, and oxidation (M) as a variable modification.

All data were reported based on 99% confidence for protein identification and determined by a false discovery rate (FDR) ≤ 0.01; each protein had at least two unique peptides. The final ratios of protein were normalized to the median average protein ratio for unequal mixes of the labeled samples. Based on relative quantitation and statistical analysis, a 1.2-fold change cutoff coupled with a Bonferroni-corrected *t*-test was applied to categorize proteins as differentially expressed, that is, proteins with label ratios > 1.20 (*p* < 0.05) were considered significantly up-regulated and those with label ratios < 0.83 (*p* < 0.05) were significantly down-regulated [45].

### Bioinformatics analysis

All identified proteins were further analyzed for functional and biological relevance. The retrieved sequences were annotated using NCBI-BLAST 2.2.30 [46] according to the datasets of non-redundant protein sequences (NR) in the National Center for Biotechnology Information (NCBI; http://www.ncbi.nlm.nih.gov). The BLAST results were extended to the functional classifications of Gene Ontology (GO; http://www.geneontology.org/) and Pathway Annotation of the Kyoto Encyclopedia of Genes and Genomes (KEGG; http://www.genome.jp/kegg/pathway.html). GO annotation was performed using Blast2GO [47]. GO enrichment analysis showed the enriched GO terms of differentially expressed proteins and computed the number of proteins for each corresponding GO term. For GO function enrichment analysis, the pathway enrichment of differentially expressed proteins in KEGG pathways was tested [7, 48].

### Determination of enzyme activity and ATP content

The total protein of each gill sample was extracted using a Protein Extraction Kit (Invent, USA), and the protein concentration was then determined using a BCA Protein Assay Kit (Merck, GER) [7]. Subsequently, NKA activity was measured using a Na^+^-K^+^-ATPase Kit (Jiancheng, CHN) according to the manufacturer’s instructions [7], and H^+^-K^+^-ATPase activity was measured following the protocol supplied by the manufacturer of the H^+^-K^+^-ATPase Kit (JianCheng, CHN). ATP content assays were performed using an ATP assay kit (Jiancheng, CHN), following the manufacturer’s instructions.

### Primary culture of branchial cells

The gill tissue was obtained from a healthy *S. argus* individual acclimated in 25‰ SW, and the culture procedure of branchial cells was described in the previous study [7, 49, 50]. In brief, harvested gill tissue was saturated with L-15 cell culture medium containing antibiotics (1000 U penicillin and 1000 U streptomycin) [7, 50]. Subsequently, the tissue was minced thoroughly with scissors, transferred to 25 cm^2^ cell culture flasks containing 8 mL of culture medium with 20% fetal bovine serum (FBS), and then incubated at 28 °C. One-half of the medium was changed every three days for two weeks. When the migrating cells formed a complete monolayer, the cells were trypsinized with a 0.25%-trypsin solution and transferred into a T25 flask (Nunc, USA) with fresh medium containing only 10% FBS, and then maintained at 28 °C [7, 49, 50]. For osmotic stress experiments, branchial cells were subjected to hypoosmotic (150 and 250 mOsmol/L) and hyperosmotic (500 and 600 mOsmol/L) media, whereas control cultures were exposed to fresh isosmotic medium (355 mOsmol/L) according to the plasma osmotic pressure of *S. argus* under 25‰ salinity. The culture media were collected 24 h after osmotic pressure challenge.

### Immunofluorescence staining of NKAα1

The localization of NKAα1 in gill tissue was determined using immunofluorescence staining. The anti-NKAα1 antibody used in this study was purchased from Abcam Company (ab76020, UK), and its specificity in *S. argus* has been verified in our previous study [6, 7]. In the present study, gill tissue samples collected from *S. argus* acclimated to different salinities (0‰, 25‰ and 50‰) were fixed in 4% paraformaldehyde at 4 °C for 16 h, dehydrated using a graded series of sucrose solutions, embedded in Tissue-Tek O.C.T (Sakura, Japan), and cut into 20-μm sections at -20 °C. Sections were mounted on poly-l-lysine-coated slides (ShiTai, CHN), incubated in 5% skim milk for 1 h at 37 °C, incubated with anti-NKAα1 antibody (1:400 dilution, Abcam, UK) in PBS containing 0.5% Triton X-100 (PBST) overnight at 4 °C, then incubated with fluorescein isothiocyanate- (FITC-) conjugated goat anti-rabbit secondary antibody (1:1000 dilution, Abcam, UK) in PBST for 4 h at the room temperature [7, 51]. Nuclei were stained with 4′,6-diamidino-2-phenylindole (DAPI, 300 nM, Sigma-Aldrich, USA) in PBS for 5 min.

Cultured branchial cells were seeded into a chamber slide (2 wells, Nunc, USA) at a density of 5 × 10^4^ cells/well in 200 μL of culture medium, and then maintained for 4 h at 28 °C. Subsequently, cells were fixed for 15 min in 4% paraformaldehyde, incubated in 5% skim milk for 0.5 h at 37 °C, incubated with primary antibody (1:400 dilution, Abcam, UK) in PBST for 4 h at 4 °C, then with FITC-conjugated goat anti-rabbit secondary antibody (1:1000 dilution, Abcam, UK) in PBST for 1.5 h at room temperature. Nuclei were stained with DAPI (300 nM, Sigma-Aldrich, USA) in PBS for 3 min.

Samples were visualized with a Zeiss LSM510 confocal laser scanning microscope (Zeiss, GER). Negative controls were incubated with normal rabbit serum (Abcam, UK).

### Measurement of Na^+^, K^+^and Cl^−^ content in culture media via ion chromatography

Culture medium was diluted with deionized water (hypotonic medium, 1:100 and 1:150 dilution, respectively; isotonic medium, 1:200 dilution; hypertonic medium, 1:250 and 1:300 dilution, respectively) and filtered with syringe filters (0.22 μm, Thermo Scientific, USA). Relative analyses were performed using a Dionex™ ICS-1500 ion chromatography system (Thermo Scientific, USA) with an AS-DV automated sampler [7].

### Statistical analysis

All data were analyzed for normality (Kolmogorov–Smirnov’s test) and homoscedasticity of variance (Levene’s test). SPSS Statistical software version 20.0 (SPSS Inc, USA) was employed for statistical analysis of data by using one-way analysis of variance (ANOVA), followed by post-hoc Tukey’s HSD tests. Data were presented as mean ± standard error of the mean (S.E.M.), and *p* < 0.05 was considered statistically significant.

## Results

### Protein identification, quantitation and functional annotation

**Fig. 2:**
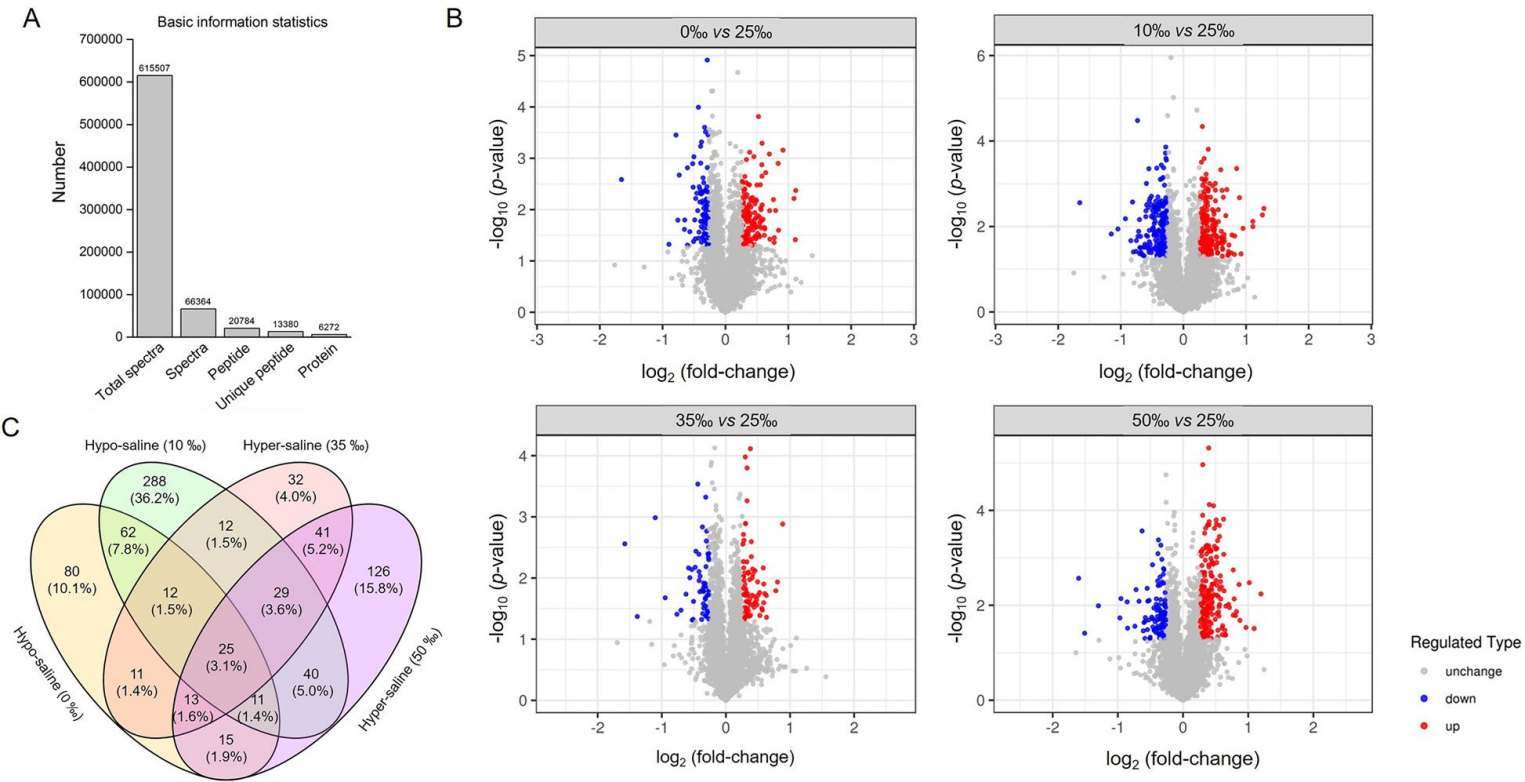
Proteomic profiles in *S. argus* gill by iTRAQ. **A** Total spectra are the secondary mass spectra, and the spectra are the secondary mass spectra after quality control. A unique peptide indicates an identified peptide belonging to only one group of proteins. Proteins were identified by Mascot 2.3.02 software. **B** Volcano plots showing the distribution of the protein expression measurements for the 6,272 quantified proteins in the gills of *S. argus* under different environmental salinity stresses, according to the -log_10_(*p*-value) of the Bonferroni-corrected *t*-test and the log_2_(fold-change) in expression between the treatment group (0‰, 10‰, 35‰ and 50‰) and the control group (25‰). Blue circles represent down-regulated proteins with a *p*-value < 0.05, red circles represent up-regulated proteins with a *p*-value < 0.05, and gray circles represent unchanged proteins. **C** Venn diagram of the substantial differences in the gill proteome of *S. argus* in response to different environmental salinities

For reducing the probability of false peptide identification, only peptides with a 95% confidence interval by Mascot probability analysis greater than ‘identity’ were finally identified and counted. For protein quantitation, a protein was required to contain at least two unique spectra. A total of 615,507 spectra were obtained from the iTRAQ-nanoLC-MS/MS proteomic analysis. Following data filtration to eliminate low-scoring spectra, 66,364 spectra were ultimately filtered to meet the strict confidence (Fig. 2A). Similar protein values (4,892, 5,165, and 5,089) were identified in each of the three replicates, resulting in a total of 6,272 proteins identified under the 1% FDR threshold at both the peptide and protein levels. Among these identified proteins, 3,897 (62.1%) were common to the three technological repeats, and 1,604 proteins containing at least two peptides were further quantitated by iTRAQ analysis (Table 1). In comparison with the control group (25‰ SW), 796 quantitated proteins meeting the screening criteria were classified as differentially expressed. Among these proteins, 741 were up-regulated and 442 were down-regulated following salinity stress (Table 1). In detail, 229, 479, 175, and 300 proteins were differentially expressed following different salinity conditions (0‰, 10‰, 35‰ and 50‰), respectively (Table 1, Fig. 2B). The numbers and percentages of proteins in Venn regions revealed substantially different responses to salinity stresses in the gills of *S. argus* (Fig. 2C).

**Table 1:** Summary statistics of gill proteome in *S. argus*

	**Hypo-saline stress**						**Control**			**Hyper-saline stress**					
Sample groups	**0 ‰—1**	**0 ‰—2**	**0 ‰—3**	**10 ‰—1**	**10 ‰—2**	**10 ‰—3**	**25 ‰—1**	**25 ‰—2**	**25 ‰—3**	**35 ‰—1**	**35 ‰—2**	**35 ‰—3**	**50 ‰—1**	**50 ‰—2**	**50 ‰—3**
The peptide number in iTRAQ protein library	**13,380**														
The number of identified proteins	**6272**														
The number and ratio of identified protein, which had been identified in all three technologic repeats	**3897 (62.1%)**														
Up-regulation (lable ratio > 1.20 and *p*-value < 0.05)	**143**			**275**			**-**			**115**			**208**		
Down-regulation (lable ratio < 0.83 and *p*-value < 0.05)	**86**			**204**			**-**			**60**			**92**		

Subsequently, Gene Ontology term analysis was performed to provide an initial assessment of the physiological processes occurring in the gills of *S. argus* during salinity stress. The UniProt databases were searched to yield the relevant information, and 796 DEPs were classified into three main GO categories: ‘biological process (BP),’ ‘cellular component (CC),’ and ‘molecular function (MF)’ (Fig. S2). KEGG is a database resource for understanding the high-level functions and utilities of proteins in biological systems, particularly from large-scale datasets [52]. To further elucidate the biological pathways involved in the reaction of the gills of *S. argus* to salinity stress, the protein sequences were mapped using KEGG pathway tools. The DEPs (796) were consequently classified into 305 specific pathways. ‘Metabolism’ and ‘Organismal systems’ were vital classifications in KEGG analysis and contained the subcategories of ‘energy metabolism’, ‘carbohydrate metabolism’ and ‘excretory system’. Among 305 mapped pathways, a total of 60 KEGG pathways were significantly enriched for DEPs (Table S1). Functional annotation analysis indicated that the physiological function of *S. argus* gills was significantly influenced by exposure to a variable salinity environment.

### Identification of environmental salinity adaptation-related DEPs

**Fig. 3:**
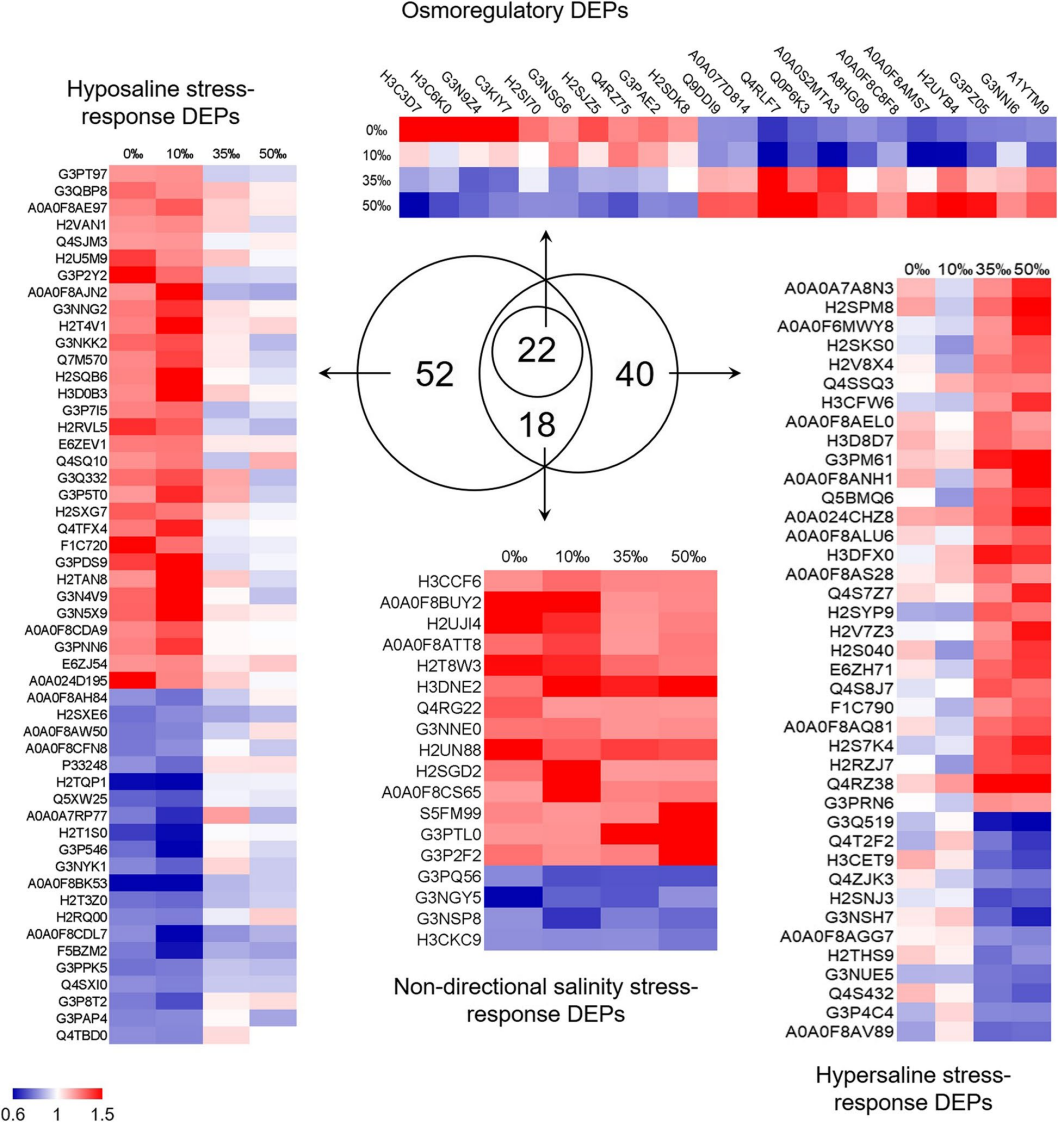
Identification of environmental salinity adaptation-related DEPs in the gills of *S. argus*. Osmoregulatory protein (22), hyposaline stress (52), hypersaline stress (40), and non-directional salinity stress (18) response DEPs were selected from the DEPs. Red represents up-regulation, and blue represents down-regulation

To investigate the differences in gill response mechanisms induced by different environmental salinity stresses, osmoregulatory, non-directional, hypo- and hypersaline stress response DEPs were identified as described by Li and Kültz [15]. In the present study, a total of 22 DEPs were consistently positively or inversely correlated with a salinity change and were considered as osmoregulatory proteins (Fig. 3, Table S2). Non-directional stress response DEPs were distinguished from osmoregulatory DEPs by their consistent occurrence during both hypo- and hypersaline stress. Eighteen non-directional stress response DEPs that were always up- or down-regulated independent of the direction of salinity change were identified (Fig. 3, Table S3). In addition, for accurately judging the difference in branchial response strategies under different salinity conditions, 52 significant DEPs due to hyposaline response and 40 significant DEPs owing to hypersaline stress were finally selected, respectively (Fig. 3, Table S4 and S5). Further analysis of these DEPs can provide a deep comprehension of osmoregulatory strategies in the gills of *S. argus* under salinity stresses.

### GO term and KEGG pathway enrichment analysis of selected DEPs

**Fig. 4:**
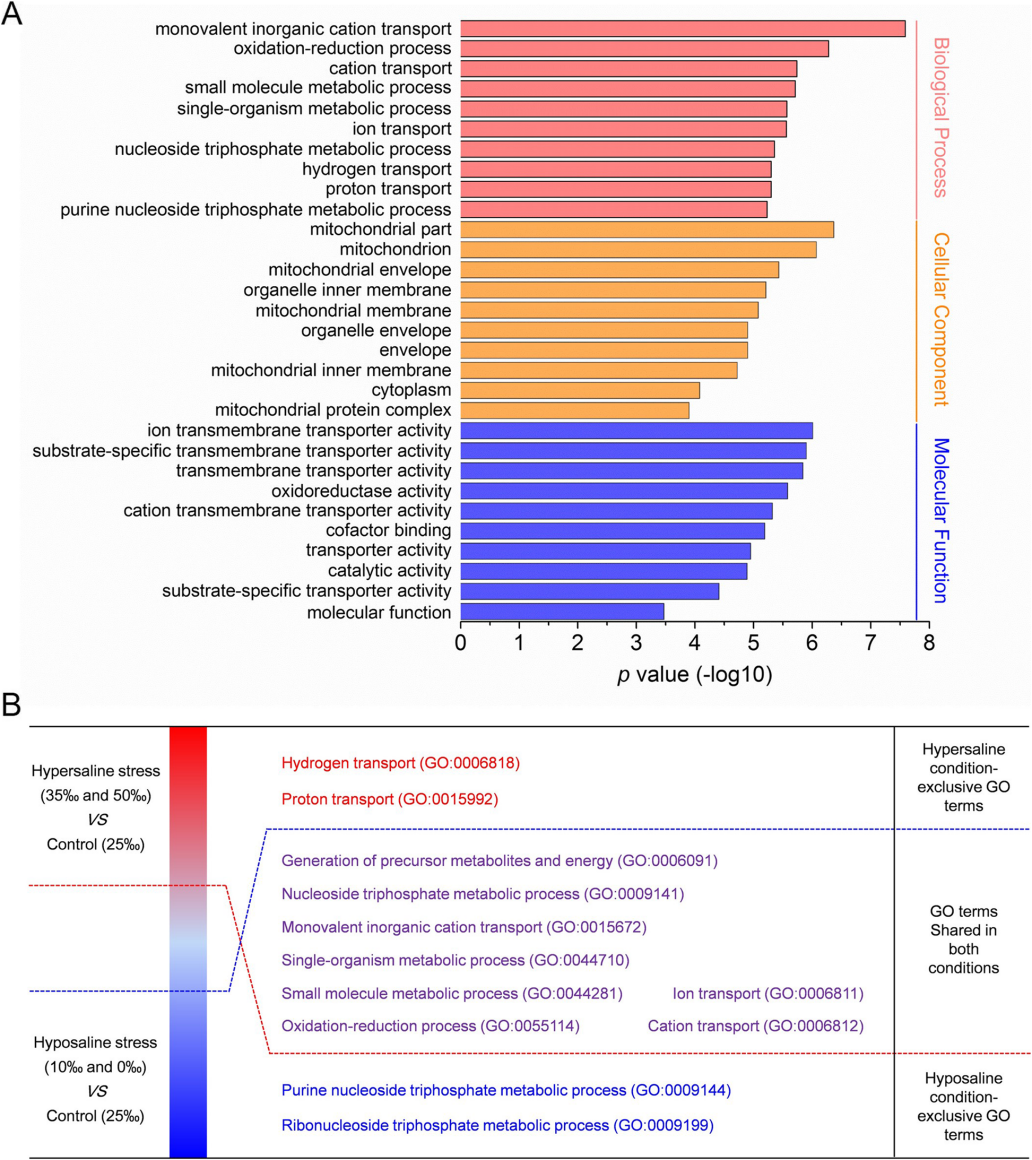
GO enrichment analysis of selected DEPs. **A** Subcategories of GO terms (*p* < 0.05, top 10) mapped with 22 proteins osmoregulatory DEPs, and **B** subcategories of GO terms (*p* < 0.05, top 10) mapped with hyposaline stress, hypersaline stress, and non-directional salinity stress DEPs in the gills of *S. argus*

**Fig. 5:**
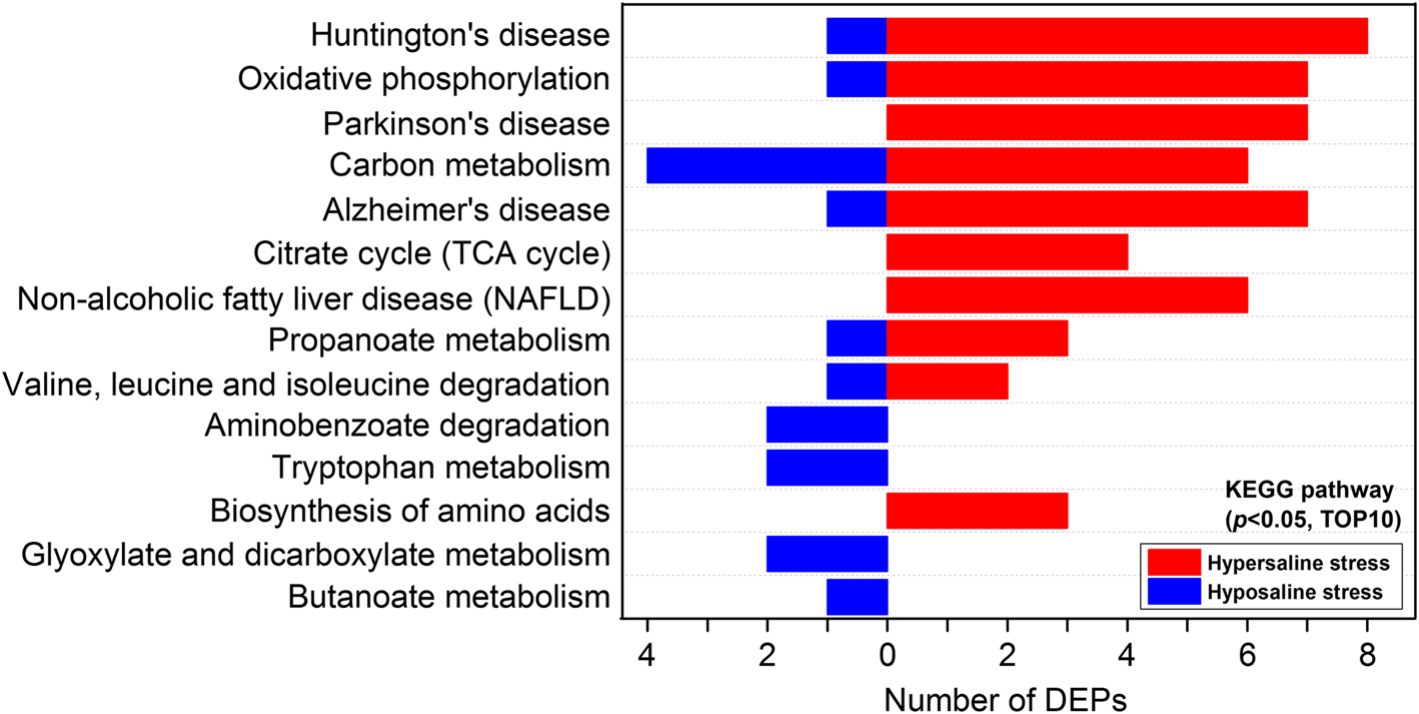
Classification of KEGG pathways (*p* < 0.05, top 10) mapped with selected DEPs in gills acclimated to different salinities. Red represents selected DEPs from hypersaline stress groups, and blue represents selected DEPs from hyposaline stress groups

The possible functional roles of selected DEPs were further analyzed by GO term and KEGG pathway annotation. The significantly enriched GO terms in osmoregulatory DEPs included ‘monovalent inorganic cation transport (GO:0,015,672)’ and ‘oxidation–reduction process (GO:0,055,114)’ (*p* < 0.05, Fig. 4A). For hyposaline, hypersaline and non-directional salinity stress-response DEPs, eight GO categories significantly annotated in both conditions included ‘generation of precursor metabolites and energy (GO:0,006,091)’, ‘monovalent inorganic cation transport (GO:0,015,672)’, ‘oxidation–reduction process (GO:0,055,114)’, etc. (*p* < 0.05, Fig. 4B). The GO terms ‘hydrogen transport (GO:0,006,818)’ and ‘proton transport (GO:0,015,992)’ were only enriched for hypersaline stress-response DEPs. The ‘Purine nucleoside triphosphate metabolic process (GO:0,009,144)’ and ‘Ribonucleoside triphosphate metabolic process (GO:0,009,199)’ were hyposaline condition-exclusive categories. Additionally, the pathways mapped by all selected DEPs were identified based on KEGG enrichment analysis. The top 10 enriched pathways with *p* < 0.05 are shown in Fig. 5. The KEGG pathways that were enriched significantly for DEPs induced by hypo- and/or hypersaline stresses included ‘oxidative phosphorylation (ko00190)’, ‘carbon metabolism (ko01200)’, ‘propanoate metabolism (ko00640)’, ‘citrate cycle (TCA cycle) (ko00020)’, and ‘valine, leucine and isoleucine degradation (ko00280)’. Interestingly, KEGG pathway enrichment analysis indicated that four human disease-related ‘Huntington's disease (ko05016)’, ‘non-alcoholic fatty liver disease (NAFLD) (ko04932)’, ‘Alzheimer's disease (ko05010)’, and ‘Parkinson's disease (ko05012)’ pathways were significantly enriched for selected DEPs (Fig. 5). Further analysis demonstrated that the DEPs mapped to these pathways (e.g. NADH dehydrogenase [ubiquinone] 1 alpha subcomplex subunit 2, NDUFA2; NADH dehydrogenase [ubiquinone] iron-sulfur protein 5, NDUFS5; Cytochrome b-c1 complex subunit Rieske, UQCRFS1; ATP synthase subunit epsilon, ATP5F1E) were mainly involved in energy metabolism. Our results suggested that the energy metabolism and ion regulation of *S. argus* gills were significantly influenced by environmental salinity, but the response strategies under variable salinity conditions were quite different.

### Expression patterns of candidate DEPs related to ion transport and energy metabolism under different salinity conditions

Based on the functional annotation results, 20 ion transport- and energy metabolism-related DEPs were identified. The differences in gill response strategy under hypo- and hypersaline stress were evaluated through the expression levels of these DEPs. Among them, 6 ion transport-related DEPs, Na^+^-K^+^-2Cl^−^ cotransporter 1 (NKCC1), voltage-dependent anion-selective channel protein 3 (VDAC3), transcription factor AP-2 beta (TFAP2B), Na^+^-K^+^-ATPase subunit alpha 1 and beta 1 (NKA α1 and β1), and H^+^-K^+^-ATPase subunit alpha (ATP4A), were detected. Their expression patterns are shown in Fig. S3. NKCC1, VDAC3 and TFAP2B were considered as osmoregulatory DEPs because their abundances were consistently positively (NKCC1 and VDAC3) or negatively (TFAP2B) correlated with salinity changes. The expression levels of NKCC1 and VDAC3 were inhibited and increased TFAP2B abundance was stimulated under hyposaline stress but showed the opposite trend under hypersaline conditions. The non-directional stress response DEP ATP4A was always up-regulated under salinity challenges. The expression levels of NKA α1 and β1 were only increased in the hypersaline stress groups. These results suggested that the gills under a hypersaline environment might possess higher ion transport activity.

Four enzymes participating in the tricarboxylic acid cycle (TCA cycle), aspartate aminotransferase (GOT2), dihydrolipoyl dehydrogenase (DLD), aconitate hydratase (ACO) and succinyl-CoA ligase [ADP-forming] subunit beta (SUCLA2) and seven enzymes of respiratory chain complex I (NDUFA2; NDUFA4; NADH-ubiquinone oxidoreductase 75 kDa subunit, NDUFS1; NDUFS5), complex III (UQCRFS1; Cytochrome b-c1 complex subunit 6, UQCRH), and complex IV (ATP5F1E) were hypersaline stress response-exclusive DEPs, which were only significantly increased in the hypersaline groups (fold-change > 1.2, Fig. S4). Additionally, another three DEPs related to ATP synthesis and transport, NAD(P) transhydrogenase (NNT), 2-oxoisovalerate dehydrogenase subunit beta (BCKDHB) and ADP-ATP translocase 2 (SLC25A5), were reduced under hyposaline stress but increased in the hypersaline groups (Fig. S4). These results implied a higher energy supply in hypersaline conditions, which might be associated with high ion transport activity under such conditions.

### Localization of NKAα1 protein in the gill of *S. argus* acclimated to different salinities

**Fig. 6:**
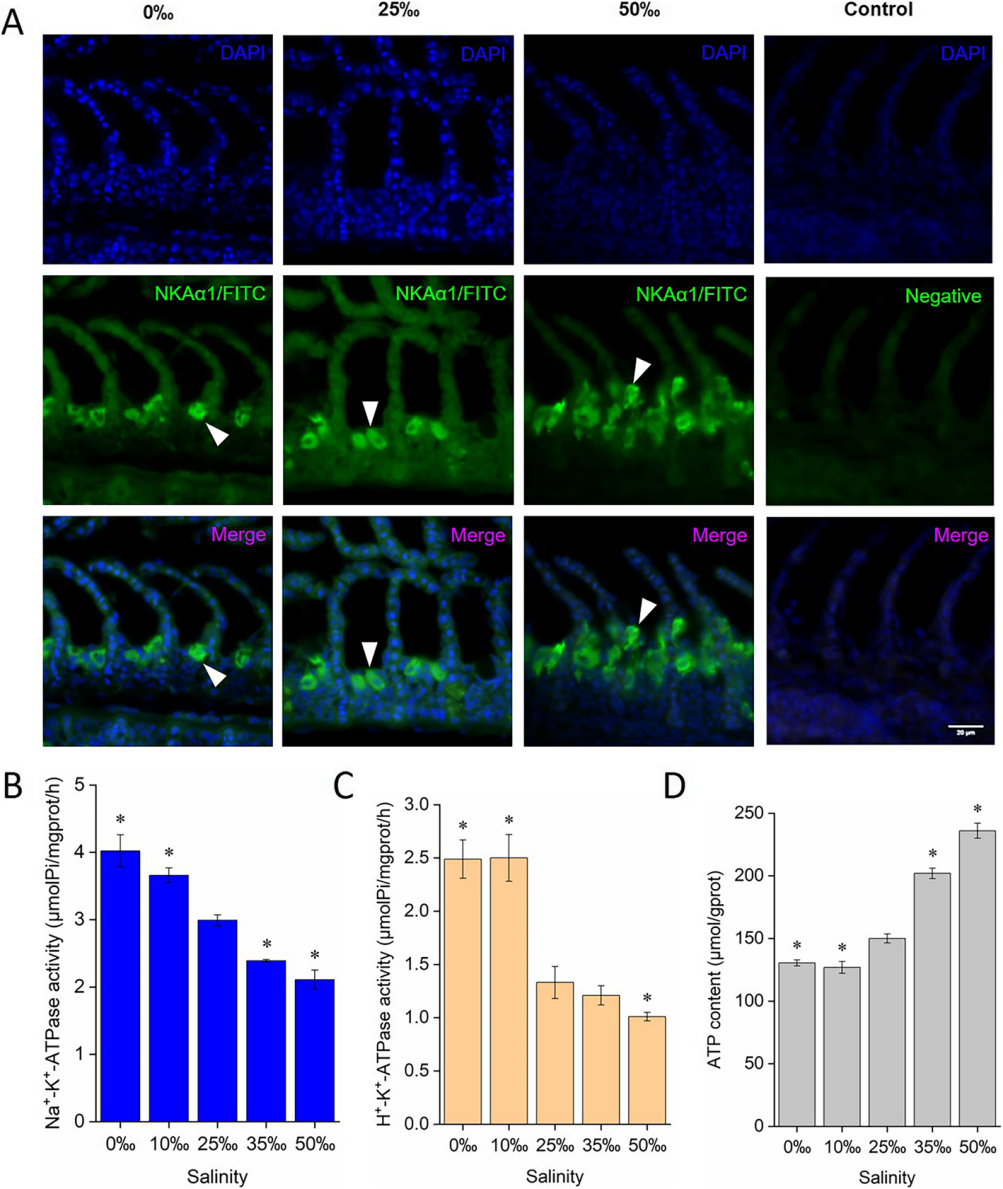
**A** Immunolocalization of NKAα1 in gills exposed to different salinity environments. NKAα1 was visualized using a primary antibody against NKAα1 (green), and nuclei were identified by staining with DAPI (blue). Negative control was incubated with normal rabbit serum. Scale bar = 20 μm. Changes in **B** NKA activity, **C** H^+^-K.^+^-ATPase activity and **D** ATP content present in gill tissues from *S. argus* acclimated to different salinities. Enzyme activity and ATP content in the gill of 25‰ SW-reared *S. argus* were used as controls. *p < 0.05

Gill sections were double-stained with primary antibody against NKAα1 (green) and DAPI (blue). The arrows in Fig. 6A indicate that NKAα1 was localized to the membranes of MRCs in fixed gill sections. Compared with the gills of *S. argus* acclimated to 0‰ and 25‰, hypersaline stress (50‰) promoted the increase of NKAα1 expression (Fig. 6A), which was consistent with the results of iTRAQ-based proteomic.

### Activities of Na^+^-K^+^-ATPase and H^+^-K^+^-ATPase, and ATP content

Compared with control treatment (NKA: 2.99 ± 0.08 μmolPi/mgprot/h; H^+^-K^+^-ATPase: 1.33 ± 0.10 μmolPi/mgprot/h), chronic exposure to hyposaline conditions significantly increased the activities of NKA (0‰: 4.02 ± 0.24 μmolPi/mgprot/h; 10‰: 3.66 ± 0.11 μmolPi/mgprot/h) and H^+^-K^+^-ATPase (0‰: 2.49 ± 0.18 μmolPi/mgprot/h; 10‰: 2.50 ± 0.22 μmolPi/mgprot/h) (*p* < 0.05) (Fig. 6B and C). In contrast, the activities of NKA (35‰: 2.39 ± 0.02 μmolPi/mgprot/h; 50‰: 2.11 ± 0.14 μmolPi/mgprot/h) and H^+^-K^+^-ATPase (50‰: 1.01 ± 0.14 μmolPi/mgprot/h) in the gills under hypersaline conditions were significantly inhibited (*p* < 0.05) (Fig. 6B and C).

Additionally, changes in the expression of critical enzymes related to TCA cycle and mitochondrial respiration ultimately affect ATP synthesis. Therefore, the concentrations of ATP *in vivo* were measured. ATP contents in the gill tissues under hyposaline conditions were significantly reduced (0‰: 130.45 ± 2.39 μmol/gprot; 10‰: 126.88 ± 4.80 μmol/gprot) compared with control group (150.04 ± 3.60 μmol/gprot) (*p* < 0.05). In contrast, the ATP contents in gills exposure to 35‰ (202.00 ± 4.20 μmol/gprot) and 50‰ (236.02 ± 6.00 μmol/gprot) SW were remarkable increased (*p* < 0.05) (Fig. 6D).

### Expression of NKAα1 in branchial cells

**Fig. 7:**
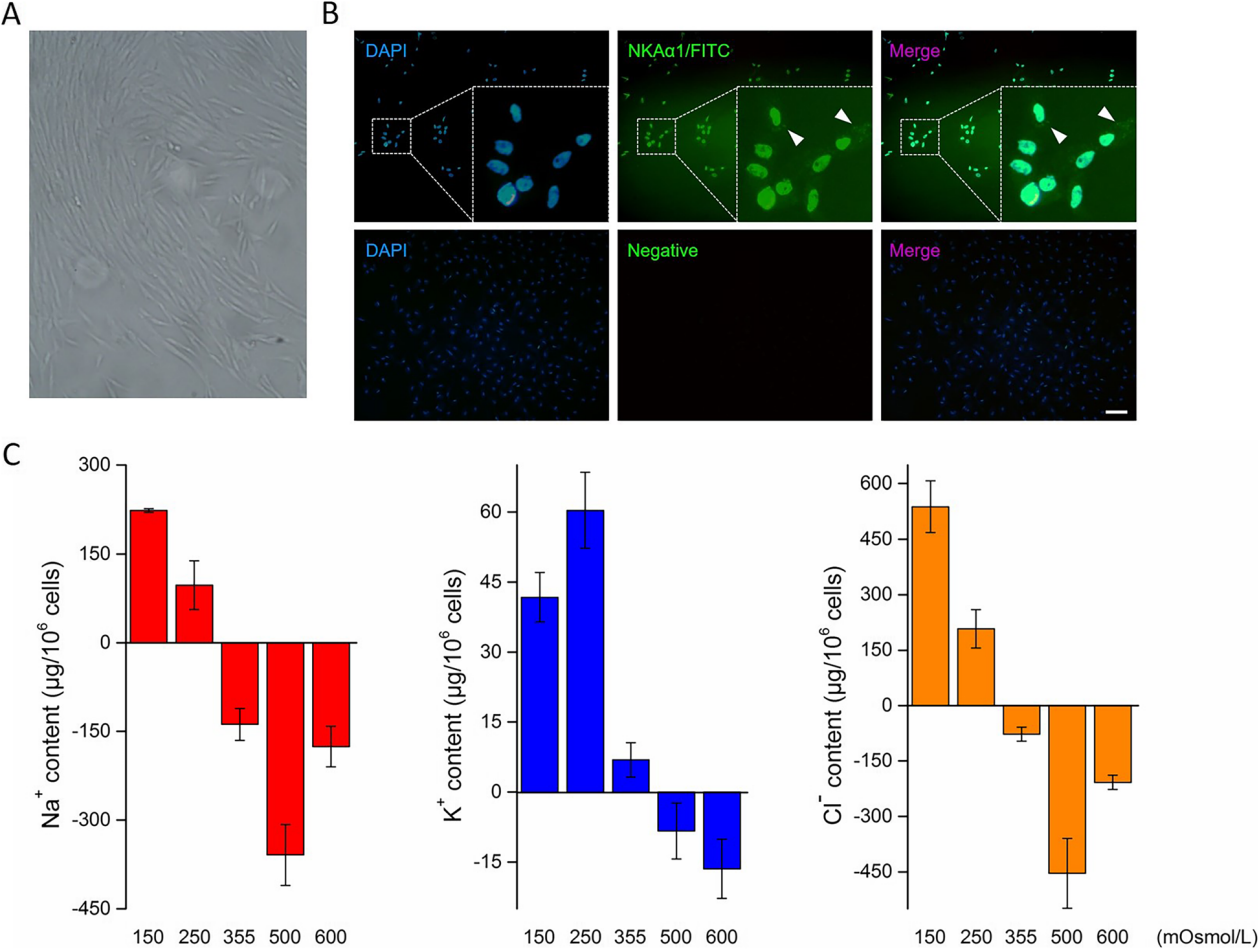
**A** The primary culture of branchial cells. **B** Immunolocalization of NKAα1 (green) in cultured branchial cells. Negative control was incubated with normal rabbit serum. Scale bar = 50 μm. **C** Analysis of Na^+^, K^+^ and Cl^−^ ion transport in branchial cells under hypo- (150 and 250 mOsmol/L) and hyperosmotic (500 and 600 mOsmol/L) stress conditions using an ion chromatography system

Immunofluorescence staining was conducted to determine the expression of NKAα1 in primary cultured cells (Fig. 7A). Branchial cells were double-stained with primary antibody against NKAα1 (green) and DAPI (blue) (Fig. 7B). This result showed NKAα1 was exclusively localized on the membranes of cultured branchial cells. No fluorescent signal was observed in negative control cell samples. Thus, our result indicated that cultured branchial cells contained abundant MRCs.

### Analysis of Na^+^, K^+^ and cl^−^ transport

The concentrations of Na^+^, K^+^ and Cl^−^ ions in culture media at different osmotic pressures were measured using ion chromatography. The changes of ion concentrations in different culture media were presented in Fig. 7C: the values greater than 0 indicate ion efflux (out of the cell), and the negative values indicate ion influx (into the cell). Na^+^ transport in branchial cells induced by osmotic stress was consistent with the Cl^−^ concentration. After exposure of branchial cells to the hypotonic medium, Na^+^ and Cl^−^ were pumped out of cells, whereas they were absorbed into cells in hypertonic medium, although the influx of Na^+^ (138.05 ± 27.16 μg/10^6^ cells) and Cl^−^ (77.40 ± 19.27 μg/10^6^ cells) was observed under isosmotic conditions. The contents of Na^+^ (223.71 ± 3.16 *vs* 97.37 ± 41.38 μg/10^6^ cells) and Cl^−^ (537.95 ± 69.61 *vs* 207.81 ± 51.92 μg/10^6^ cells) excreted by branchial cells in 150 mOsmol/L medium were over twice than outflowed under 250 mOsmol/L condition. In contrast, exposure of branchial cells to hypertonic medium promoted Na^+^ and Cl^−^ influx to maintain the homeostasis. Especially, the influx of Na^+^ ion (358.85 ± 51.54 *vs* 175.68 ± 34.41 μg/10^6^ cells) and Cl^−^ ion (453.72 ± 94.42 *vs* 207.70 ± 19.4 μg/10^6^ cells) in 500 mOsmol/L medium was approximately twice than in 600 mOsmol/L medium. In addition, K^+^ ion was pumped out of branchial cells during hypotonic stress (150 mOsmol/L: 41.75 ± 5.30 μg/10^6^ cells; 250 mOsmol/L: 60.37 ± 8.09 μg/10^6^ cells), although an influx of K^+^ ion was observed under hypertonic conditions (500 mOsmol/L: 8.32 ± 5.98 μg/10^6^ cells; 600 mOsmol/L: 16.43 ± 6.33 μg/10^6^ cells). Our results demonstrated that Na^+^, K^+^ and Cl^−^ ions were pumped out of branchial cells under hyposaline conditions and were absorbed into cells under hypersaline stresses.

## Discussion

In recent years, *S. argus* has emerged as a new mariculture species in South China [5]. The farming of this species is mainly restricted to coastal net cages located in areas with a wide variation in salinity [53]. Frequent salinity changes as an environmental perturbation can greatly influence growth, reproduction and development of fish, often affecting the economic benefits of mariculture to some extent [2, 54]. Thus, an understanding of osmoregulatory strategies of *S. argus* may provide valuable insights for environmental adaption, promoting the expansion of commercial aquaculture.

Fish gills are directly exposed to the external milieu and responsible for many critical physiological processes such as respiration and osmoregulation, representing an excellent target for analyzing environmental effects on fish [15, 55]. Fish gills require efficient response mechanisms dealing with frequent changes in salinity, which as an environmental perturbation can greatly affect physiologic function of estuarine fish gills [56]. Dysfunction of gill osmoregulation can cause fish to become unable to adapt to changes in environmental salinity, and thus result in death. Therefore, adaptive regulation to maintain gill osmoregulatory function is critically important for fish living in variable aquatic environments [57]. In the present study, the differential expression profiles of the gill proteome in response to hypo- and hypersaline stress in *S. argus* were elucidated. To ensure the accuracy of our results, two salinity gradients were set for the hypo- (0‰ and 10‰) and hypersaline (35‰ and 50‰) stress groups, respectively. In total, 796 DEPs were identified by the iTRAQ-based comparative proteomic analysis. Among these DEPs, 22 osmoregulatory proteins and 18 non-directional stress response proteins were ultimately identified. Additionally, 52 hyposaline stress response-exclusive and 40 hypersaline stress response-exclusive DEPs were also selected to explore the distinction of salinity adaptive response mechanisms in the gill under different salinity environments. Bioinformatics analysis of relative DEPs revealed that energy metabolism and ion regulatory function in the gills of *S. argus* were significantly affected by salinity stress (*p* < 0.05, Figs. 4 and 5).

Effective ion transport in fish gills under salinity stress is based on a lot of ion pumps, channels and exchangers [26, 28, 39]. Our results showed that the expression levels of proteins involved in ion transport (NKA α1 and β1, NKCC1, ATP4A, VDAC3, and TFAP2B) were significantly changed under salinity stress (fold-change > 1.2 or < 0.83, *p* < 0.05). NKCC and NKA are important biomarkers in branchial MRCs which is the major cell type in fish gills that actively transport ions [22]. Generally, NKCC is regarded as an important ion transporter under salinity stress in fish [30, 34, 36]. Basolateral NKCC is highly expressed in branchial cells under hyperosmotic conditions; in contrast, low levels of branchial NKCC expression are associated with FW acclimation [28, 37, 58]. In the present study, NKCC was tightly related with osmoregulation, and its expression was suppressed when salinity decreased (0‰: 0.75-fold; 10‰: 0.74-fold) and elevated with increased salinity (35‰: 1.29-fold; 50‰: 2.07-fold) (Fig. S3). This means that hypersaline stress induces high expression of NKCC to promote Na^+^, K^+^ and Cl^−^ ion reabsorption, which is inhibited under low salinity conditions, thus reducing the osmotic pressure gradient between the inside and outside of the cell.

Accumulating evidences have indicated that the ion gradients reduced by basolateral NKCC are generated by the action of NKA [25]. Na^+^ exit from the cell is ignited by basolateral NKA which is commonly regarded as a vital osmoregulatory protein [22]. The protein abundance of NKAα1 is generally supposed to be up-regulated with increased salinity and decreased when salinity drops in euryhaline fish [28, 37]. However, in *Poecilia latipinna* and *Lateolabrax maculatus*, it has been proven that the expression level of NKAα1 increases in the hypersaline condition and remains stable when salinity decreases [32, 38]. In the present study, NKAα1 was localized to branchial MRCs (Fig. 6A), and the expression levels of NKAα1 were only significantly up-regulated (> 1.2-fold, Fig. S3) in the gill of *S. argus* acclimated to hypertonic conditions which were associated with the results of immunofluorescence staining (Fig. 6A). The α-subunit of NKA is a catalytic subtype that cleaves high-energy phosphate bonds and exchanges intracellular Na^+^ ion for extracellular K^+^ ion and is predominantly expressed in the gill [59]. Elevated branchial NKAα1 in hypersaline-acclimated fish stimulated excess salt efflux, which is necessary to avoid excessive intracellular osmotic pressure. The β-subunit is a glycosylated transmembrane protein that regulates assembly of the α/β heterodimer and its insertion into the plasma membrane, requiring normal NKA function [60]. Previous studies also reported an increase of NKA β1 in eels after transferring to hypersaline environment [61]. Over-expression of NKAβ1 can enhance NKA function [62, 63]. Thus, increased branchial NKAβ1 expression in *S. argus* exposed to high salinity (> 1.2-fold, Fig. S3) could stimulate the action of NKA, exacerbating salt excretion. In *Chanos chanos* exposed to FW, it has been also proved that the protein expression of gill NKAβ1 were significantly increased within 198 h [64]. Therefore, we speculate that long-term freshwater stress can induce the increase of NKAβ1 protein expression in the gills but only in initial stages. Additionally, the α1/β1 combination is considered as the predominant heterodimer of gills participating in primary ion uptake or secretion [65]. Parallel expression of NKA α1 and β1 were often observed in gills of fish [64]. Similar to NKAα1, the similar expression profiles of NKAβ1 in the gill of *S. argus* increased in the hypertonic conditions and remains stable when salinity decreases (Fig. S3). Our results showed that the enzyme activity of branchial NKA was decreased under hypersaline treatment and increased in hyposaline environments (Fig. 6B). Additionally, NKA function is also influenced by internalization of the catalytic a-subunits mediated by transcription factor adaptor protein 2 (TFAP2) [66]. Cell-surface NKA levels are sensitive to changes in the extracellular environment, which can rapidly drive the internalization of the NKA from the cell membrane to the cytoplasm, which can inhibit Na^+^ secretion mediated by NKA [66]. The expression of TFAP2B in *S. argus* gills was increased under hyposaline conditions and reduced under hypersaline stress (Fig. S3), suggesting that TFAP2B-induced NKA internalization was enhanced to prevent excessive Na^+^ loss of branchial cells in low salinity environments but inhibited under high salinity stresses. On that account, we suspect that NKA might be involved in the high salinity response in *S. argus* gills by affecting the expression of the catalytic subtype and anchoring the enzyme complex in membranes, which promoted excess Na^+^ excretion, and NKA activity was suppressed as a compensatory regulation. Under hyposaline conditions, the relatively stable expression of NKAα1 and β1 and elevated NKA activity could maintain the normal level of Na^+^ excretion to reduce the osmotic pressure of cells, thus promoting adaptation to the hypotonic environment. Previous studies also found that the expression levels of NKA was inconsistent with its activity, which might be related to post-translational modifications [67, 68]. Meanwhile, NKA internalization was also required to prevent excessive Na^+^ loss in a hyposaline environment. This ion regulatory strategy addresses the unique salinity adaption in *S. argus*.

Collaborating with the basolateral NKA, epithelial sodium channel (ENaC) expressed in the apical membrane regulates salt influx and plays a major role in the control of internal salt balance, which can be inhibited by VDAC3 [69, 70]. In the gill of *S. argus* exposed to different environmental salinities, the abundance of ENaC remained relatively stable throughout the experimental course. However, elevated branchial VDAC3 in hypersaline-acclimated *S. argus* enhanced the inhibition of Na^+^ influx through ENaC, whereas hyposaline stresses weakened this function of VDAC3 by reducing its expression. This strategy mediated by VDAC3 is supposed to regulate the entry of external Na^+^ into cells through ENaC, maintaining intracellular Na^+^ concentration stability. In addition, the expression level of ATP4A, the catalytic subunit of ion pump H^+^-K^+^-ATPase with a cation and ATP binding site, was significantly increased (> 1.2-fold) under both hypo- and hypersaline stresses (Fig. S3). Similar to NKA, the enzyme activity of H^+^-K^+^-ATPase was increased in gills exposed to hyposaline conditions and inhibited under hypersaline treatment (Fig. 6C). ATP4A is responsible for H^+^ excretion in exchange for K^+^ ion at the expense of ATP consumption in cells [71]. To our knowledge, K^+^ ion is an essential osmolyte for maintaining a high intracellular osmolarity in the cell [72]. Therefore, elevated ATP4A and altered enzyme activity were essential for increasing intracellular osmotic pressure under salinity stress.

Active ion shifts between intra- and extracellular compartments mediated by some ion transporters (e.g. NKA and ATP4A) under salinity stress have been demonstrated to be involved with the highly energy-consuming process [73, 74]. Energy metabolism for osmoregulation is a research hotspot in fish physiology, which extremely affects the energy allocation in fish growth and reproduction and then decreases the economic benefits [73]. The TCA cycle and mitochondrial respiration are crucial pathways for cellular ATP synthesis [75, 76]. In the present study, four important mitochondrial enzymes associated with TCA cycle (GOT2, DLD, ACO and SUCLA2) of branchial cells also presented over-expression (fold-change > 1.2) under hypertonic conditions (Fig. S4). In general, the TCA cycle begins with the condensation of acetyl moiety of acetyl-CoA with oxaloacetate to form citrate, which is isomerized to isocitrate by mitochondrial ACO and then decarboxylated to α-ketoglutarate [77]. GOT2 also catalyzes the synthesis of α-ketoglutarate, which is the initial step for ATP production in the TCA cycle [78, 79]. The synthesis of α-ketoglutarate from succinate is catalyzed by SUCLA2 [80, 81]. However, stimulation of DLD can reduce α-ketoglutarate but increase succinate production [82], revealing that DLD up-regulation induced by high salinity stress in branchial cells may increase downstream metabolites of TCA cycle and promote mitochondrial ATP production. BCKDHB participates in α-ketoglutarate production in the process of energy generation [83, 84]. In this study, an obvious elevation of BCKDHB was observed under hypersaline conditions (> 1.2-fold), while its expression levels were significantly reduced after hyposaline acclimation (< 0.83-fold) (Fig. S4). The remarkable up-regulation of BCKDHB indicates that branched-chain amino acids are also being more heavily utilized to provide the TCA cycle with a substrate. Otherwise, TCA cycle intermediates are replenished through the mediation of GOT2 route [79]. Interestingly, TCA intermediate routes mediated by BCKDHB and GOT2 in the gills of hypersaline-acclimated *S. argus* were both enhanced, promoting ATP synthesis to power the cells quickly. As is well known, complex I (NADH–ubiquinone oxidoreductase), complex II (succinate–quinone oxidoreductase), complex III (cytochrome b-c1), complex IV (cytochrome c oxidase) and complex V (ATP synthase) in the mitochondrial respiratory chain can generate proton motive force that in turn drives ATP synthesis [85]. Our results revealed that hypersaline stress significantly increased the expression of several subunits of mitochondrial complex I (NDUFA2, NDUFA4, NDUFS1 and NDUFS5), complex III (UQCRFS1, UQCRH) and complex V (ATP5F1E) (Fig. S4). These results indicate that more energy is needed and efficient ATP synthesis in branchial cells under hypersaline environments.

A high level of energy consumption can result in the formation of reactive oxygen species (ROS) that contribute to oxidative stress directly [86]. NTT, identified as a protective protein, was positively correlated with environmental salinity changes (Fig. S4, Table S2), which can generate NADPH, a cofactor required for antioxidant-related enzyme activity [87]. Synthetic ATP within mitochondria is transported out to the cytosol, while ADP recruitment is accomplished through SLC25A5, thus playing an essential role in the supply of mitochondrial ATP to the cytoplasm [88]. Elevated expression of SLC25A5 in the gills of high salinity-acclimated *S. argus* promoted ATP export across the mitochondrial inner membrane to the cytoplasm; in turn, the activity of ATP export was reduced when SLC25A5 expression was inhibited by hyposaline stress. The results of ATP content detection also demonstrated that ATP synthesis in the gills acclimated to hyposaline conditions was suppressed, whereas it was enhanced under hypersaline treatments (Fig. 6C). Therefore, high expression levels of these proteins could enhance the energy supply of branchial cells during hypersaline adaptation, indicating higher energy demand than that under hypotonic stress.

Modulation of ion transporter expression and energy production are directly associated with the exchange of intra- and extracellular ions. In the present study, primary branchial cells were used to analyze ion transport under different salinity environments. An abundance of MRCs, the specific ionocytes involved in active ion transport, in cultured branchial cells were determined by immunofluorescence localization of NKAα1 (Fig. 7B). In cultured cells exposed to hypoosmotic medium, K^+^ ion was excreted to reduce intracellular osmolarity, whereas it was ingested into cells under hyperosmotic stress (Fig. 7C). Although K^+^ ion plays a vital role in maintaining the osmolality of intracellular and extracellular fluids, Na^+^ and Cl^−^ ions are the two most dominant types of ions related to osmotic balance in fish [72, 89]. In the present study, branchial cells excreted Na^+^ and Cl^−^ ions under hypoosmotic shock, whereas the influx of Na^+^ and Cl^−^ was induced by hyperosmotic stress (Fig. 7C), with the aim to maintain intracellular ion homeostasis. It deserves attention that Na^+^ and Cl^−^ influx in branchial cells in 500 mOsmol/L medium were approximately twice those in cells in 600 mOsmol/L medium. We suppose that the difference in Na^+^ and Cl^−^ influx between these two hyperosmotic groups may be associated with more K^+^ influx (16.43 ± 6.33 μg/10^6^ cells *vs* 8.32 ± 5.98 μg/10^6^ cells) under 600 mOsmol/L condition. It has been proven that high K^+^ concentrations are related to the increase of membrane permeability and Na^+^ loss in previous studies [90, 91]. The results above indicate that the exposure of branchial cells to hyperosmotic conditions can arouse an alternative way of ion excretion for avoiding the excessive increase intracellular osmotic pressure.

## Conclusion

**Fig. 8:**
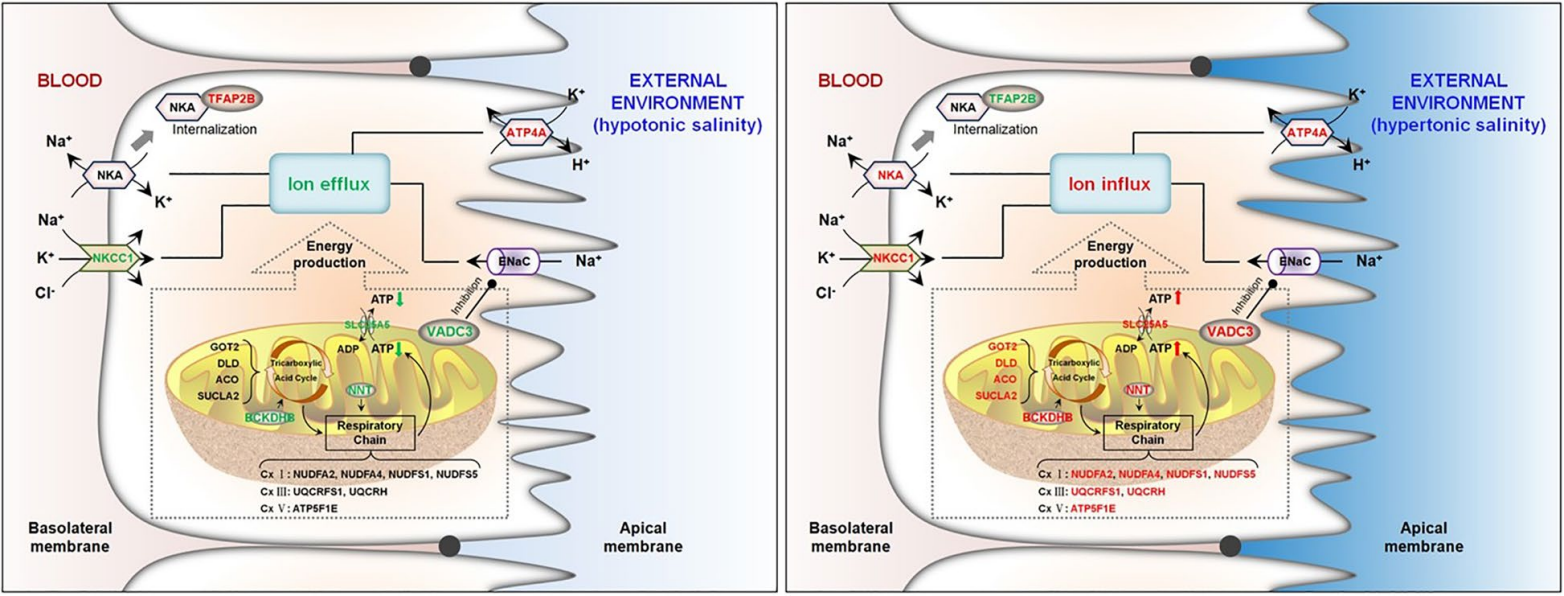
The deduced model of osmoregulatory strategies in the gills of *S. argus* responding to variable environmental salinity

Analysis of proteome profiles under different salinity conditions indicate divergent osmoregulatory strategies of *S. argus* responding to hypo- and hypersaline environments (Fig. 8). Proper Na^+^ efflux was maintained through elevated NKA activity during hyposaline stress, which was conducive to reducing the intracellular osmotic pressure. Simultaneously, increased NKA internalization and reduced energy supply in cells were ignited to prevent excessive loss of ions. Under the hypersaline condition, excess ions entering cells along the salinity gradient were excreted through active transport with sufficient mitochondrial energy. Enhanced NKCC1-mediated Na^+^ reabsorption and reduced NKA activity were considered as a compensatory regulation to appropriately increase intracellular osmotic pressure. In summary, the gills under different salinity stresses efficiently regulate the content of intracellular ions dependent on diverse osmoregulatory strategies, facilitating rapid adaptation to different environmental salinity, which is essential for *S. argus* to possess a high tolerance to a wide range of salinities. This study provides new insights into the osmoregulatory mechanisms and environmental adaption of estuarine fish.

## Supplementary Information

**Additional file 1: Table S1.** List of the significantly enriched GO terms for quantitated proteins. **Table S2.** List of the osmoregulatory proteins in the gills of *Scatophagus argus *during salinity challenge. **Table S3.** List of the non-directional salinity-stress response proteins in the gills of *Scatophagus argus *during salinity challenge. **Table S4.** List of the hyposaline-stress response proteins in the gills of *Scatophagus argus *during salinity challenge. **Table S5.** List of the hypersaline-stress response proteins in the gills of *Scatophagus argus *during salinity challenge. **Fig. S1.** The body weight of *S. agrus *exposed to different salinities. **Figure S2.** GO classification of DEPs. The results for ‘biological process (BP)’, ‘cellular component (CC)’ and ‘molecular function (MF)’ terms were summarized. **Figure S3.** Expression levels of six DEPs related to ion transport in the gills of *S. argus *exposed to different salinity environments identified by iTRAQ technology. The red dotted line represents a significant up-regulation with a threshold of 1.2-fold, and the blue dotted line represents a significant down-regulation with a threshold of 0.83-fold. Blue circles represent fold change < 0.83, red circles represent fold change > 1.2, and black circles represent 0.83 < fold change < 1.2. **Figure S4.** Expression levels of 14 DEPs related to energy metabolism in the gills of *S. argus *exposed to different salinity environments identified by iTRAQ technology. The red dotted line represents a significant up-regulation with a threshold of 1.2-fold, and the blue dotted line represents a significant down-regulation with a threshold of 0.83-fold. Blue circles represent fold change < 0.83, red circles represent fold change > 1.2, and black circles represent 0.83 < fold change < 1.2.

## Data Availability

The datasets used and/or analysed during the current study available from the corresponding author on reasonable request.

## Abbreviations

iTRAQ: Isobaric tags for relative and absolute quantitation
LC–MS/MS: Liquid chromatography-tandem mass spectrometry
DEP: Differentially expressed protein
NKA: Na^+^-K^+^-ATPase
NKCC: Na^+^-K^+^-2Cl^−^ cotransporter
ATP: Adenosine triphosphate
SW: Seawater
FW: Freshwater
MS-222: 3-Aminobenzoic acid ethyl ester methanesulfonate
SCX: Strong cation exchange
HCD: High-energy collision-induced dissociation
CID: Collision-induced dissociation
pAGC: Predictive automatic gain control
FDR: False discovery rate
NR: Non-redundant protein sequences
NCBI: National Center for Biotechnology Information
GO: Gene Ontology
KEGG: Pathway Annotation of the Kyoto Encyclopedia of Genes and Genomes
FBS: Fetal bovine serum
PBST: PBS containing 0.5% Triton X-100
FITC: Fluorescein isothiocyanate
DAPI: 4′,6-Diamidino-2-phenylindole
ANOVA: One-way analysis of variance
BP: Biological process
CC: Cellular component
MF: Molecular function
NDUFA2: NADH dehydrogenase [ubiquinone] 1 alpha subcomplex subunit 2
NDUFS5: NADH dehydrogenase [ubiquinone] iron-sulfur protein 5
UQCRFS1: Cytochrome b-c1 complex subunit Rieske
ATP5F1E: ATP synthase subunit epsilon
VDAC3: Voltage-dependent anion-selective channel protein 3
TFAP2B: Transcription factor AP-2 beta
ATP4A: H^+^/K^+^ ATPase subunit alpha
TCA cycle: Tricarboxylic acid cycle
GOT2: Aspartate aminotransferase
DLD: Dihydrolipoyl dehydrogenase
ACO: Aconitate hydratase
SUCLA2: Succinyl-CoA ligase [ADP-forming] subunit beta
NDUFS1: NADH-ubiquinone oxidoreductase 75 kDa subunit
UQCRH: Cytochrome b-c1 complex subunit 6
NNT: NAD(P) transhydrogenase
BCKDHB: 2-Oxoisovalerate dehydrogenase subunit beta
SLC25A5: ADP-ATP translocase 2
ENaC: Epithelial sodium channel
