# Supplementary material for: Osmoregulatory strategies of estuarine fish Scatophagus argus in response to environmental salinity changes
Source: BMC Genomics. 2022 Jul 30;23:545. doi: 10.1186/s12864-022-08784-2 (PMC9339187; doi:10.1186/s12864-022-08784-2)
Supplement: Supplementary file 1 — Additional file 1: Table S1. List of the significantly enriched GO terms for quantitated proteins. Table S2. List of the osmoregulatory proteins in the gills of Scatophagus argus during salinity challenge. Table S3. List of the non-directional salinity-stress response proteins in the gills of Scatophagus argus during salinity challenge. Table S4. List of the hyposaline-stress response proteins in the gills of Scatophagus argus during salinity challenge. Table S5. List of the hypersaline-stress response proteins in the gills of Scatophagus argus during salinity challenge. Fig. S1. The body weight of S. agrus exposed to different salinities. Figure S2. GO classification of DEPs. The results for ‘biological process (BP)’, ‘cellular component (CC)’ and ‘molecular function (MF)’ terms were summarized. Figure S3. Expression levels of six DEPs related to ion transport in the gills of S. argus exposed to different salinity environments identified by iTRAQ technology. The red dotted line represents a significant up-regulation with a threshold of 1.2-fold, and the blue dotted line represents a significant down-regulation with a threshold of 0.83-fold. Blue circles represent fold change < 0.83, red circles represent fold change > 1.2, and black circles represent 0.83 < fold change < 1.2. Figure S4. Expression levels of 14 DEPs related to energy metabolism in the gills of S. argus exposed to different salinity environments identified by iTRAQ technology. The red dotted line represents a significant up-regulation with a threshold of 1.2-fold, and the blue dotted line represents a significant down-regulation with a threshold of 0.83-fold. Blue circles represent fold change < 0.83, red circles represent fold change > 1.2, and black circles represent 0.83 < fold change < 1.2. [file 12864_2022_8784_MOESM1_ESM.pdf]

Osmoregulatory strategies of estuarine fish S*catophagus argus* in response to environmental salinity changes

Maoliang Su, Nanxi Liu, Zhengqi Zhang, Junbin Zhang*

Shenzhen Key Laboratory of Marine Bioresource & Eco-Environmental Science, College of Life Sciences and Oceanography, Shenzhen University, Shenzhen 518060, China

* Corresponding author: Junbin Zhang

E-mail: jbzhang@szu.edu.cn

Tel: (+86 755) 2653 6629

Table S1. List of the significantly enriched GO terms for quantitated proteins

**Map ID**	**Pathway name**	**Number of enriched proteins**	***p*-value**
ko05016	Huntington's disease	75	9.99E-10
ko00190	Oxidative phosphorylation	60	1.77E-09
ko05012	Parkinson's disease	62	1.61E-07
ko01200	Carbon metabolism	80	3.93E-07
ko05010	Alzheimer's disease	66	1.64E-06
ko00020	Citrate cycle (TCA cycle)	31	2.23E-06
ko04932	Non-alcoholic fatty liver disease (NAFLD)	49	6.22E-06
ko00640	Propanoate metabolism	24	1.68E-05
ko00280	Valine, leucine and isoleucine degradation	34	1.74E-05
ko00930	Caprolactam degradation	7	7.09E-05
ko00627	Aminobenzoate degradation	6	0.0003
ko00380	Tryptophan metabolism	16	0.0003
ko01230	Biosynthesis of amino acids	42	0.0003
ko00630	Glyoxylate and dicarboxylate metabolism	21	0.0004
ko00650	Butanoate metabolism	12	0.0005
ko00010	Glycolysis / Gluconeogenesis	43	0.0010
ko00710	Carbon fixation in photosynthetic organisms	20	0.0011
ko00062	Fatty acid elongation	10	0.0029
ko01210	2-Oxocarboxylic acid metabolism	12	0.0041
ko04972	Pancreatic secretion	24	0.0052
ko04974	Protein digestion and absorption	29	0.0060
ko00071	Fatty acid degradation	24	0.0064
ko04610	Complement and coagulation cascades	25	0.0074
ko00310	Lysine degradation	13	0.0088
ko01212	Fatty acid metabolism	21	0.0090
ko00900	Terpenoid backbone biosynthesis	6	0.0096
ko00072	Synthesis and degradation of ketone bodies	6	0.0096
ko04971	Gastric acid secretion	19	0.0098
ko00620	Pyruvate metabolism	23	0.0099
ko04964	Proximal tubule bicarbonate reclamation	13	0.0119
ko04973	Carbohydrate digestion and absorption	12	0.0120

ko00030	Pentose phosphate pathway	18	0.0128
ko05130	Pathogenic Escherichia coli infection	24	0.0132
ko04210	Apoptosis	31	0.0152
ko04931	Insulin resistance	24	0.0154
ko00760	Nicotinate and nicotinamide metabolism	9	0.0158
ko04978	Mineral absorption	10	0.0163
ko00330	Arginine and proline metabolism	17	0.0165
ko03010	Ribosome	33	0.0170
ko04011	MAPK signaling pathway - yeast	13	0.0201
ko00720	Carbon fixation pathways in prokaryotes	12	0.0210
ko04976	Bile secretion	12	0.0210
ko00480	Glutathione metabolism	16	0.0213
ko04922	Glucagon signaling pathway	25	0.0221
ko04961	Endocrine and other factor-regulated calcium reabsorption	15	0.0225
ko04260	Cardiac muscle contraction	28	0.0233
ko05100	Bacterial invasion of epithelial cells	31	0.0265
ko04960	Aldosterone-regulated sodium reabsorption	10	0.0293
ko04142	Lysosome	24	0.0301
ko00970	Aminoacyl-tRNA biosynthesis	18	0.0309
ko00950	Isoquinoline alkaloid biosynthesis	4	0.0355
ko05134	Legionellosis	22	0.0385
ko04966	Collecting duct acid secretion	9	0.0396
ko04066	HIF-1 signaling pathway	24	0.0413
ko01040	Biosynthesis of unsaturated fatty acids	6	0.0438
ko05412	Arrhythmogenic right ventricular cardiomyopathy (ARVC)	20	0.0442
ko04144	Endocytosis	54	0.0483
ko00281	Geraniol degradation	3	0.0497
ko00960	Tropane, piperidine and pyridine alkaloid biosynthesis	3	0.0497
ko00400	Phenylalanine, tyrosine and tryptophan biosynthesis	3	0.0497

Table S2. List of the osmoregulatory proteins in the gills of *Scatophagus argus* during salinity challenge

Accession	Abbreviation	Protein description	Fold change				Organism
			0‰	10‰	35‰	50‰	
Q0P6K3	NKCC1	Na^{+}-K^{+}-2Cl^{-} cotransporter 1	0.754	0.742	1.285	2.073	*Dicentrarchus labrax*
H2UYB4	VDAC3	Voltage-dependent anion-selective channel protein 3	0.762	0.605	1.271	1.842	*Takifugu rubripes*
G3PZ05	NNT	NAD(P) transhydrogenase, mitochondrial	0.796	0.735	1.102	1.471	*Gasterosteus aculeatus*
G3NNI6	BCKDHB	2-oxoisovalerate dehydrogenase subunit beta, mitochondrial	0.801	0.952	1.134	1.225	*Gasterosteus aculeatus*
A1YTM9	HPX	Hemopexin	0.816	0.743	1.234	1.329	*Dicentrarchus labrax*
A0A0F8C8F8	CP	Ceruloplasmin	0.796	0.864	1.160	1.205	*Larimichthys crocea*
A0A0F8AMS7	SLA	Snaclec bitiscetin subunit alpha	0.728	0.558	1.015	1.442	*Larimichthys crocea*
A0A0S2MTA3	MHCIIβ	MHC class II antigen beta chain	0.793	0.599	1.416	1.387	*Scatophagus argus*
A8HG09	SLC25A5	ADP-ATP translocase 2	0.828	0.745	1.003	1.335	*Epinephelus coioides*
Q9DDI9	TF	Serotransferrin	0.826	0.823	1.160	1.329	*Ammodytes marinus*
A0A077D814	SOD2	Superoxide dismutase	0.822	0.856	1.150	1.322	*Larimichthys crocea*
Q4RLF7	SRP68	Signal recognition particle subunit SRP68	0.679	0.341	2.345	1.814	*Tetraodon nigroviridis*
H3C3D7	TES	Testin	1.529	1.085	0.857	0.518	*Tetraodon nigroviridis*
H3C6K0	EPX	Eosinophil peroxidase	1.967	0.957	0.896	0.718	*Tetraodon nigroviridis*
G3N9Z4	SAMHD1	Deoxynucleoside triphosphate triphosphohydrolase SAMHD1	2.124	1.041	0.748	0.758	*Gasterosteus aculeatus*
C3KIY7	NUDC	Nuclear migration protein nudC	2.044	1.091	0.771	0.802	*Anoplopoma fimbria*

H2SI70	MMP13	Collagenase 3	1.278	1.002	0.975	0.819	*Takifugu rubripes*
G3NSG6	VWA5A	von Willebrand factor A domain-containing protein 5A	1.209	1.248	0.817	0.819	*Gasterosteus aculeatus*
H2SJZ5	TUBB5	Tubulin beta-5 chain	1.349	1.047	0.878	0.773	*Takifugu rubripes*
Q4RZ75	TFAP2B	Transcription factor AP-2-beta	1.231	1.260	0.872	0.726	*Tetraodon nigroviridis*
G3PAE2	PLXNA2	Plexin-A2	1.276	1.171	0.902	0.813	*Gasterosteus aculeatus*
H2SDK8	DKC1	H/ACA ribonucleoprotein complex subunit DKC1	1.204	1.049	1.001	0.803	*Takifugu rubripes*

Table S3. List of the non-directional salinity-stress response proteins in the gills of *Scatophagus argus* during salinity challenge

**Accession**	**Abbreviation**	**Protein description**	**Fold change**				**Organism**
			**0‰**	**10‰**	**35‰**	**50‰**	
H3CCF6	ALDH4A1	Delta-1-pyrroline-5-carboxylate dehydrogenase, mitochondrial	1.218	1.294	1.238	1.235	*Tetraodon nigroviridis*
A0A0F8BUY2	PTGES2	Prostaglandin E synthase 2	2.999	1.558	1.214	1.234	*Larimichthys crocea*
H2UJI4	GNG10	Guanine nucleotide-binding protein G(I)/G(S)/G(O) subunit gamma-10	1.601	1.421	1.206	1.250	*Takifugu rubripes*
A0A0F8ATT8	IARS2	Isoleucine--tRNA ligase, mitochondrial	1.287	1.376	1.203	1.261	*Larimichthys crocea*
H2T8W3	HSPG2	Basement membrane-specific heparan sulfate proteoglycan core protein	1.481	1.427	1.292	1.256	*Takifugu rubripes*
H3DNE2	ZFR	Zinc finger RNA-binding protein	1.279	1.882	1.442	1.575	*Tetraodon nigroviridis*
Q4RG22	PPPα2	Serine/threonine-protein phosphatase alpha-2	1.331	1.203	1.210	1.205	*Tetraodon nigroviridis*
G3P2F2	ATP4A	H^+/K^+ ATPase subunit alpha	1.277	1.219	1.251	1.551	*Gasterosteus aculeatus*
G3NNE0	KLHL41	Kelch-like protein 41	1.272	1.281	1.210	1.224	*Gasterosteus aculeatus*

H2UN88	MYH(F)	Myosin heavy chain, fast skeletal muscle	2.202	1.317	1.381	1.356	*Takifugu rubripes*
H2SGD2	NME	Nucleoside diphosphate kinase	1.273	1.619	1.201	1.202	*Takifugu rubripes*
A0A0F8CS65	ARID5B	AT-rich interactive domain-containing protein 5B	1.200	1.933	1.245	1.259	*Larimichthys crocea*
S5FM99	COX2	Cytochrome c oxidase subunit 2	1.238	1.209	1.228	1.540	*Scatophagus argus*
G3PTL0	TRADD	Tumor necrosis factor receptor type 1-associated DEATH domain protein	1.211	1.212	1.625	1.627	*Gasterosteus aculeatus*
G3PQ56	MYH11	Myosin-11	0.772	0.552	0.523	0.472	*Gasterosteus aculeatus*
G3NGY5	GAPR1	Golgi-associated plant pathogenesis-related protein 1	0.491	0.754	0.735	0.826	*Gasterosteus aculeatus*
G3NSP8	FKBP1A	Peptidylprolyl isomerase	0.829	0.680	0.800	0.763	*Gasterosteus aculeatus*
H3CKC9	EPPK1	Epiplakin	0.825	0.818	0.821	0.787	*Tetraodon nigroviridis*

Table S4. List of the hyposaline-stress response proteins in the gills of *Scatophagus argus* during salinity challenge

**Accession**	**Abbreviation**	**Protein description**	**Fold change**				**Organism**
			**0‰**	**10‰**	**35‰**	**50‰**	
G3PT97	USP10	Ubiquitin carboxyl-terminal hydrolase 10	1.207	1.219	0.924	0.939	*Gasterosteus aculeatus*
G3QBP8	PYGL	Alpha-1,4 glucan phosphorylase	1.293	1.224	1.128	1.036	*Gasterosteus aculeatus*
A0A0F8AE97	GNAQ	Guanine nucleotide-binding protein G(Q) subunit alpha	1.241	1.327	1.093	1.042	*Larimichthys crocea*
H2VAN1	TGM2	TGc domain-containing protein	1.213	1.233	1.095	0.943	*Takifugu rubripes*
Q4SJM3	CTNNA1	Catenin alpha-1	1.202	1.208	0.981	1.030	*Tetraodon nigroviridis*
H2U5M9	GDE	Glycogen debranching enzyme	1.385	1.227	1.118	0.990	*Takifugu rubripes*

G3P2Y2	ACAT2	Acetyl-CoA acetyltransferase, cytosolic	2.852	1.293	0.930	0.937	*Gasterosteus aculeatus*
A0A0F8AJN2	COPA	Coatomer subunit alpha	1.211	1.553	0.883	0.863	*Larimichthys crocea*
G3NNG2	VPS37B	Vacuolar protein sorting-associated protein 37B	1.262	1.405	1.064	1.020	*Gasterosteus aculeatus*
H2T4V1	HSP70	Stress-70 protein, mitochondrial	1.247	1.702	1.051	1.086	*Takifugu rubripes*
G3NKK2	NSF	Vesicle-fusing ATPase	1.301	1.352	1.039	0.892	*Gasterosteus aculeatus*
Q7M570	DES	Desmin	1.234	1.372	1.044	0.929	*Takifugu rubripes*
H2SQB6	MPP1	55 kDa erythrocyte membrane protein	1.240	1.639	1.013	0.957	*Takifugu rubripes*
H3D0B3	SND1	Staphylococcal nuclease domain-containing protein 1	1.228	1.574	1.101	1.018	*Tetraodon nigroviridis*
G3P7I5	RAP2B	Ras-related protein Rap-2b	1.247	1.289	0.892	0.950	*Gasterosteus aculeatus*
H2RVL5	EXOC2	Exocyst complex component 2	1.413	1.327	0.936	0.888	*Takifugu rubripes*
E6ZEV1	ALPL	Alkaline phosphatase, tissue-nonspecific isozyme	1.258	1.266	1.042	1.039	*Dicentrarchus labrax*
Q4SQ10	HK3	Hexokinase 3	1.218	1.260	0.909	1.158	*Tetraodon nigroviridis*
G3Q332	PRRC1	Protein PRRC1	1.283	1.351	1.173	0.897	*Gasterosteus aculeatus*
G3P5T0	COPS7A	COP9 constitutive photomorphogenic homolog subunit 7A	1.203	1.425	1.164	0.925	*Gasterosteus aculeatus*
H2SXG7	CKM	Creatine kinase M-type	1.323	1.267	1.068	0.982	*Takifugu rubripes*
Q4TFX4	PTRH2	Peptidyl-tRNA hydrolase 2, mitochondrial	1.263	1.445	0.976	1.005	*Tetraodon nigroviridis*
F1C720	HYOU1	Hypoxia up-regulated protein 1	1.495	1.283	0.962	0.974	*Perca flavescens*
G3PDS9	FDPS	Farnesyl pyrophosphate synthase	1.378	2.162	0.947	0.985	*Gasterosteus aculeatus*
H2TAN8	SEC24A	Protein transport protein Sec24A	1.213	1.514	1.106	0.942	*Takifugu rubripes*
G3N4V9	ACSL3	long-chain-fatty-acid--CoA ligase 3	1.304	1.542	1.012	0.903	*Gasterosteus aculeatus*

G3N5X9	PRPH	Peripherin	1.306	1.547	1.065	1.035	*Gasterosteus aculeatus*
A0A0F8CDA9	EPRS1	Bifunctional glutamate/proline--tRNA ligase	1.235	1.334	1.011	0.997	*Larimichthys crocea*
G3PNN6	RBBP7	Histone-binding protein RBBP7	1.246	1.394	1.011	1.007	*Gasterosteus aculeatus*
E6ZJ54	MRPL15	39S ribosomal protein L15, mitochondrial	1.212	1.236	1.051	1.109	*Dicentrarchus labrax*
A0A024D195	C3	Complement component C3	1.620	1.240	1.095	0.991	*Larimichthys crocea*
A0A0F8AH84	IGHM	Ig mu chain C region membrane-bound form / immunoglobulin heavy chain	0.828	0.777	0.918	1.027	*Larimichthys crocea*
H2SXE6	PPA	Inorganic pyrophosphatase	0.777	0.818	0.858	0.892	*Takifugu rubripes*
A0A0F8AW50	RAB8A	Ras-related protein Rab-8A	0.788	0.809	0.926	1.060	*Larimichthys crocea*
P33248	TYB12	Thymosin beta-12	0.827	0.764	1.059	1.064	*Lateolabrax japonicus*
H2TQP1	THRAP3	Thyroid hormone receptor-associated protein 3	0.612	0.555	0.975	0.979	*Takifugu rubripes*
Q5XW25	CAT	Catalase	0.756	0.732	0.980	0.962	*Oplegnathus fasciatus*
A0A0A7RP77	CA2	Red blood cell carbonic anhydrase II	0.795	0.666	1.192	0.885	*Sciaenops ocellatus*
A0A0F8CFN8	HINT1	Histidine triad nucleotide-binding protein 1	0.771	0.676	0.873	0.910	*Larimichthys crocea*
H2T1S0	KRT17	Keratin, type I cytoskeletal 17	0.695	0.614	0.995	0.986	*Takifugu rubripes*
G3P546	APOB	Apolipoprotein B-100	0.771	0.591	1.028	0.939	*Gasterosteus aculeatus*
G3NYK1	PON2	Serum paraoxonase/arylesterase 2	0.811	0.751	1.081	0.918	*Gasterosteus aculeatus*
A0A0F8BK53	CALR	Calreticulin	0.563	0.534	0.889	0.919	*Larimichthys crocea*
H2T3Z0	TAF2N	TATA-binding protein-associated factor 2N	0.779	0.764	0.893	0.926	*Takifugu rubripes*
H2RQ00	KRT13	Keratin, type I cytoskeletal 13	0.807	0.797	0.972	1.095	*Takifugu rubripes*
A0A0F8CDL7	CORO1C	Coronin 1C	0.821	0.516	0.831	0.879	*Larimichthys crocea*

F5BZM2	APOA4	Apolipoprotein A-IV1	0.792	0.626	0.871	0.851	*Epinephelus bruneus*
G3PPK5	CA	Carbonic anhydrase	0.779	0.793	0.909	0.895	*Gasterosteus aculeatus*
Q4SXI0	RCN1	Reticulocalbin-1 isoform X1	0.821	0.800	0.915	0.914	*Tetraodon nigroviridis*
G3P8T2	ISOC2	Isochorismatase domain-containing protein 2	0.795	0.721	1.035	1.068	*Gasterosteus aculeatus*
G3PAP4	SNAP23	Synaptosomal-associated protein 23	0.807	0.816	1.014	0.860	*Gasterosteus aculeatus*
Q4TBD0	NECTIN2	Nectin-2	0.821	0.812	1.070	1.001	*Tetraodon nigroviridis*

Table S5. List of the hypersaline-stress response proteins in the gills of *Scatophagus argus* during salinity challenge

**Accession**	**Abbreviation**	**Protein description**	**Fold change**				**Organism**
			**0‰**	**10‰**	**35‰**	**50‰**	
A0A0A7A8N3	NKAα1	Sodium/potassium-transporting ATPase subunit alpha 1	1.136	0.942	1.223	1.429	*Scatophagus argus*
H2SPM8	NKAβ1	Sodium/potassium-transporting ATPase subunit beta 1	1.184	0.918	1.289	1.832	*Takifugu rubripes*
A0A0F6MWY8	NDUFA2	NADH dehydrogenase [ubiquinone] 1 alpha subcomplex subunit 2	0.971	0.937	1.213	1.473	*Sparus aurata*
H2SKS0	GOT2	Aspartate aminotransferase, mitochondrial	0.957	0.831	1.220	1.333	*Takifugu rubripes*
H2V8X4	PRDX3	Thioredoxin-dependent peroxide reductase, mitochondrial	1.029	0.872	1.265	1.327	*Takifugu rubripes*
Q4SSQ3	NDUFS5	NADH dehydrogenase [ubiquinone] iron-sulfur protein 5	1.014	1.150	1.242	1.233	*Tetraodon nigroviridis*
H3CFW6	DLD	Dihydrolipoyl dehydrogenase	0.934	0.907	1.211	1.414	*Tetraodon nigroviridis*
A0A0F8AEL0	MYBPC1	Myosin-binding protein C, slow-type	1.121	1.008	1.302	1.200	*Larimichthys crocea*
H3D8D7	DPT	Dermatopontin	1.149	1.042	1.296	1.229	*Tetraodon nigroviridis*

G3PM61	UQCRFS1	Cytochrome b-c1 complex subunit Rieske, mitochondrial	1.111	1.079	1.456	1.554	*Gasterosteus aculeatus*
A0A0F8ANH1	SUCLA2	Succinyl--CoA ligase [ADP-forming] subunit beta, mitochondrial	1.160	0.903	1.225	1.549	*Larimichthys crocea*
Q5BMQ6	WAP65	Warm temperature acclimation-related 65 kDa protein	1.004	0.836	1.305	1.400	*Acanthopagrus schlegelii*
A0A024CHZ8	CTSD	Cathepsin D	1.169	1.188	1.311	1.880	*Sparus aurata*
A0A0F8ALU6	ACO	Aconitate hydratase, mitochondrial	1.085	0.977	1.251	1.325	*Larimichthys crocea*
H3DFX0	HBS1L	HBS1-like protein	0.978	1.123	1.463	1.403	*Tetraodon nigroviridis*
A0A0F8AS28	EPRS	Glutamyl-tRNA synthetase	1.041	1.119	1.290	1.204	*Larimichthys crocea*
Q4S7Z7	ATP5F1E	ATP synthase subunit epsilon, mitochondrial	1.080	1.020	1.214	1.442	*Tetraodon nigroviridis*
H2SYP9	ACADM	Medium-chain specific acyl-CoA dehydrogenase, mitochondrial	0.868	0.859	1.322	1.269	*Takifugu rubripes*
H2V7Z3	UQCRH	Cytochrome b-c1 complex subunit 6	0.987	0.995	1.220	1.466	*Takifugu rubripes*
H2S040	ITIH3	Inter-alpha-trypsin inhibitor heavy chain H3	1.118	0.835	1.248	1.399	*Takifugu rubripes*
E6ZH71	NDUFA4	NADH dehydrogenase [ubiquinone] 1 alpha subcomplex subunit 4	1.053	0.921	1.303	1.392	*Dicentrarchus labrax*
Q4S8J7	LOC106607311	Cytolysin RTX-A	0.954	0.999	1.338	1.279	*Tetraodon nigroviridis*
F1C790	SHBG	Sex hormone-binding globulin	0.984	0.881	1.212	1.304	*Perca flavescens*
A0A0F8AQ81	NDUFS1	NADH-ubiquinone oxidoreductase 75 kDa subunit, mitochondrial	1.069	0.926	1.287	1.342	*Larimichthys crocea*
H2S7K4	SNCG	Gamma-synuclein	0.916	0.960	1.341	1.445	*Takifugu rubripes*
H2RZJ7	IDH2	Isocitrate dehydrogenase [NADP], mitochondrial	0.999	0.839	1.341	1.380	*Takifugu rubripes*
Q4RZ38	FBLN1	Fibulin-1	1.090	1.199	1.520	1.684	*Tetraodon nigroviridis*
G3PRN6	GPX4	Phospholipid hydroperoxide glutathione peroxidase	1.004	0.916	1.219	1.200	*Gasterosteus aculeatus*
G3NUE5	MYG1	UPF0160 protein MYG1, mitochondrial	0.883	0.885	0.788	0.769	*Gasterosteus aculeatus*

G3P4C4	STRN4	Striatin-4	0.882	1.081	0.813	0.811	*Gasterosteus aculeatus*
A0A0F8AV89	CYTH	CYTH domain-containing protein	0.851	1.046	0.761	0.771	*Larimichthys crocea*
H2SNJ3	DCTN1	Dynactin subunit 1	0.956	0.975	0.717	0.738	*Takifugu rubripes*
G3Q519	LPCAT4	lysophospholipid acyltransferase LPCAT4	0.900	1.013	0.631	0.505	*Gasterosteus aculeatus*
A0A0F8AGG7	GOLGB1	Golgin subfamily B member 1	1.023	1.042	0.828	0.808	*Larimichthys crocea*
Q4S432	UBI	polyubiquitin	1.136	1.025	0.784	0.740	*Tetraodon nigroviridis*
H2THS9	PPP4R2	Serine/threonine-protein phosphatase 4 regulatory subunit 2	1.103	1.028	0.774	0.827	*Takifugu rubripes*
Q4T2F2	ANO1	Anoctamin 1	0.876	1.112	0.788	0.686	*Tetraodon nigroviridis*
H3CET9	GGA1	ADP-ribosylation factor-binding protein GGA1	1.159	1.045	0.758	0.705	*Tetraodon nigroviridis*
Q4ZJK3	NAMPT	Nicotinamide phosphoribosyltransferase	1.049	0.923	0.809	0.782	*Tetraodon nigroviridis*
G3NSH7	ILF3	Interleukin enhancer-binding factor 3	1.043	1.111	0.757	0.647	*Gasterosteus aculeatus*

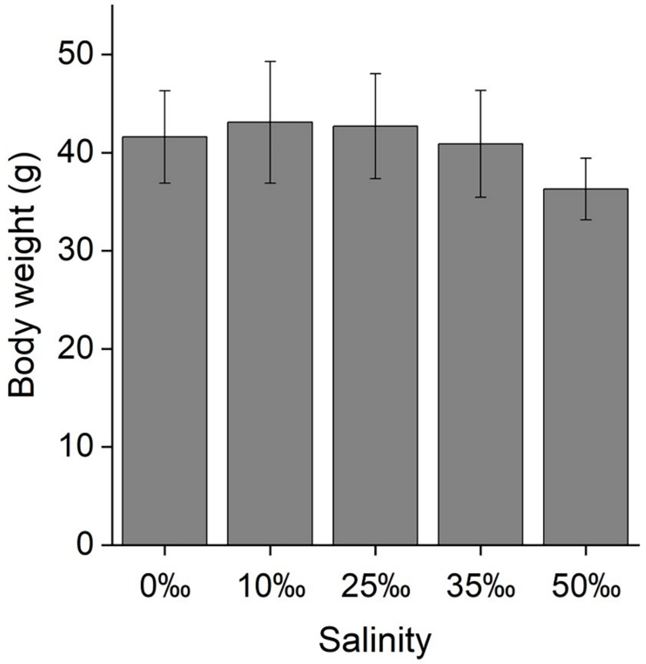

Fig. S1. The body weight of *S. agrus* exposed to different salinities.

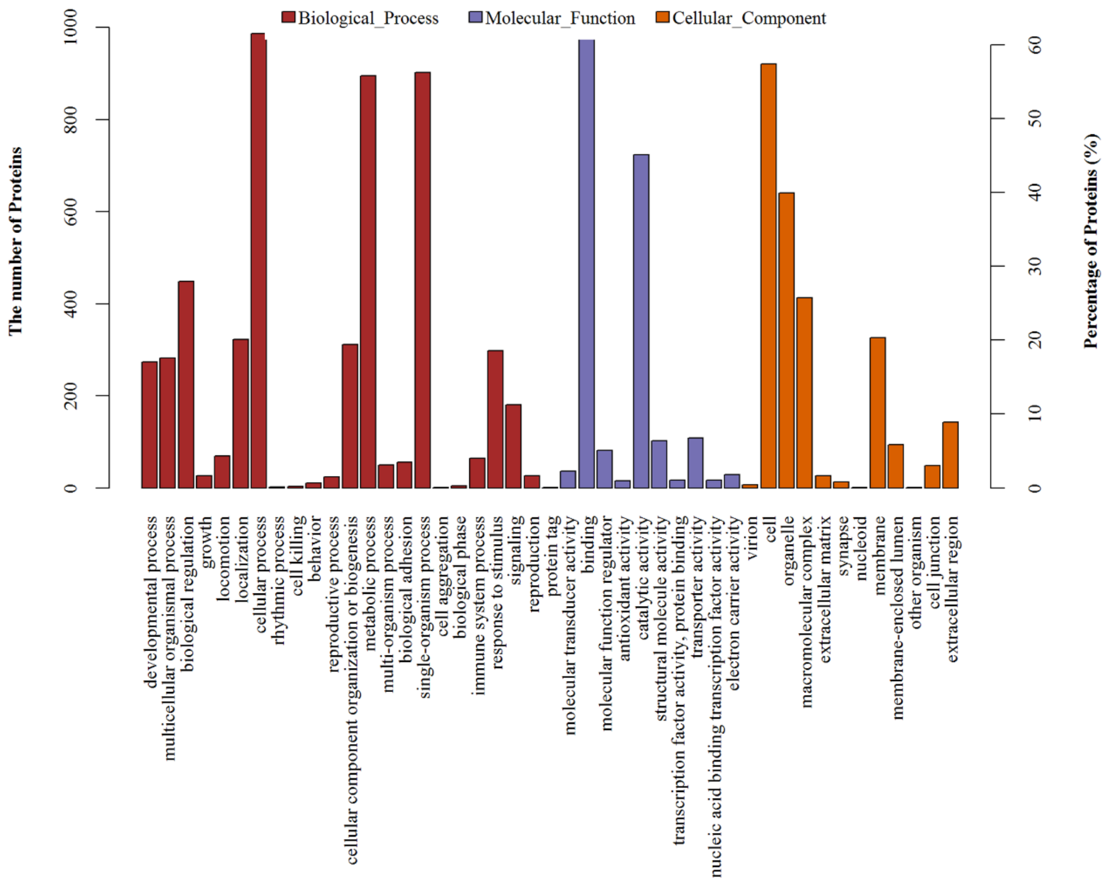

Figure S2. GO classification of DEPs. The results for ‘biological process (BP)’, ‘cellular component (CC)’ and ‘molecular function (MF)’ terms were summarized.

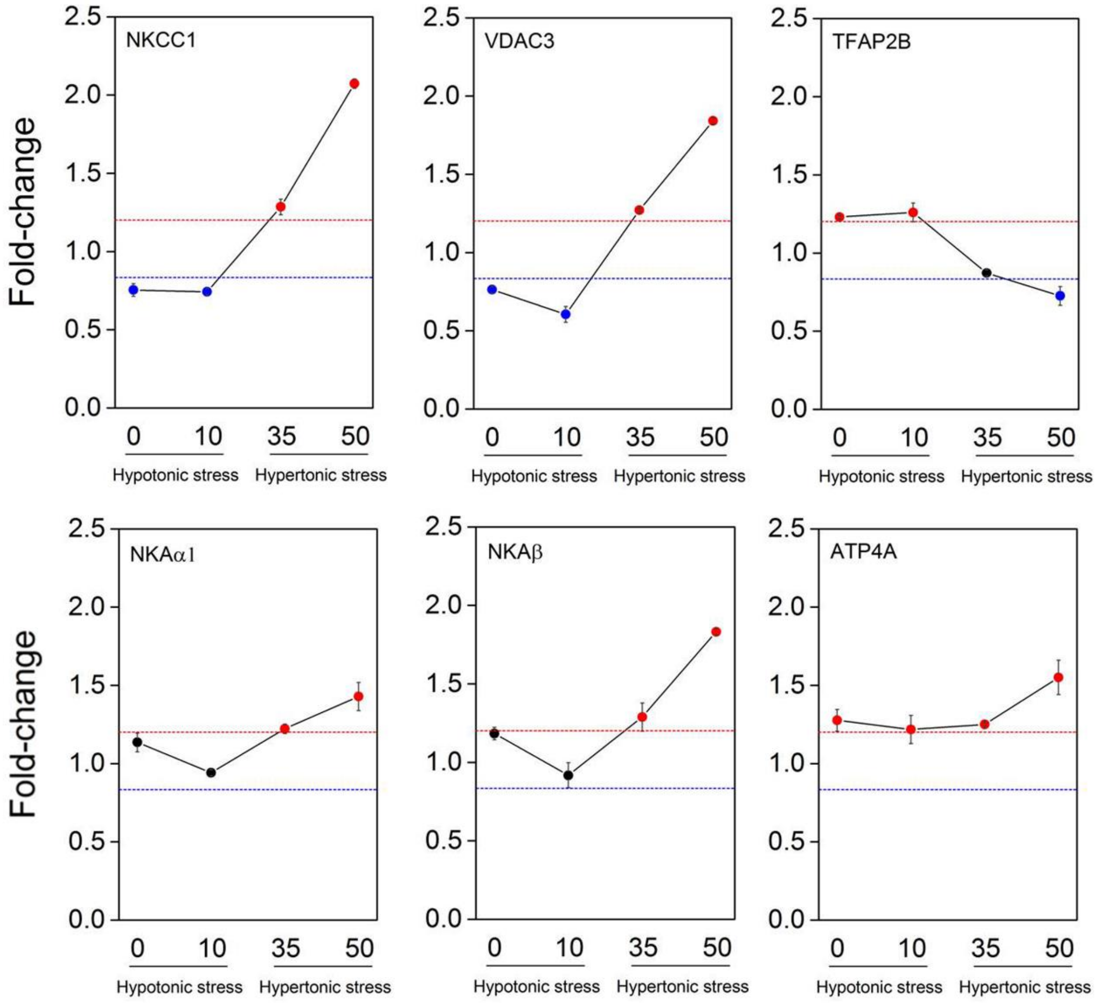

Figure S3. Expression levels of six DEPs related to ion transport in the gills of *S. argus* exposed to different salinity environments identified by iTRAQ technology. The red dotted line represents a significant up-regulation with a threshold of 1.2-fold, and the blue dotted line represents a significant down-regulation with a threshold of 0.83-fold. Blue circles represent fold change < 0.83, red circles represent fold change > 1.2, and black circles represent $0.83 <$ fold change < 1.2.

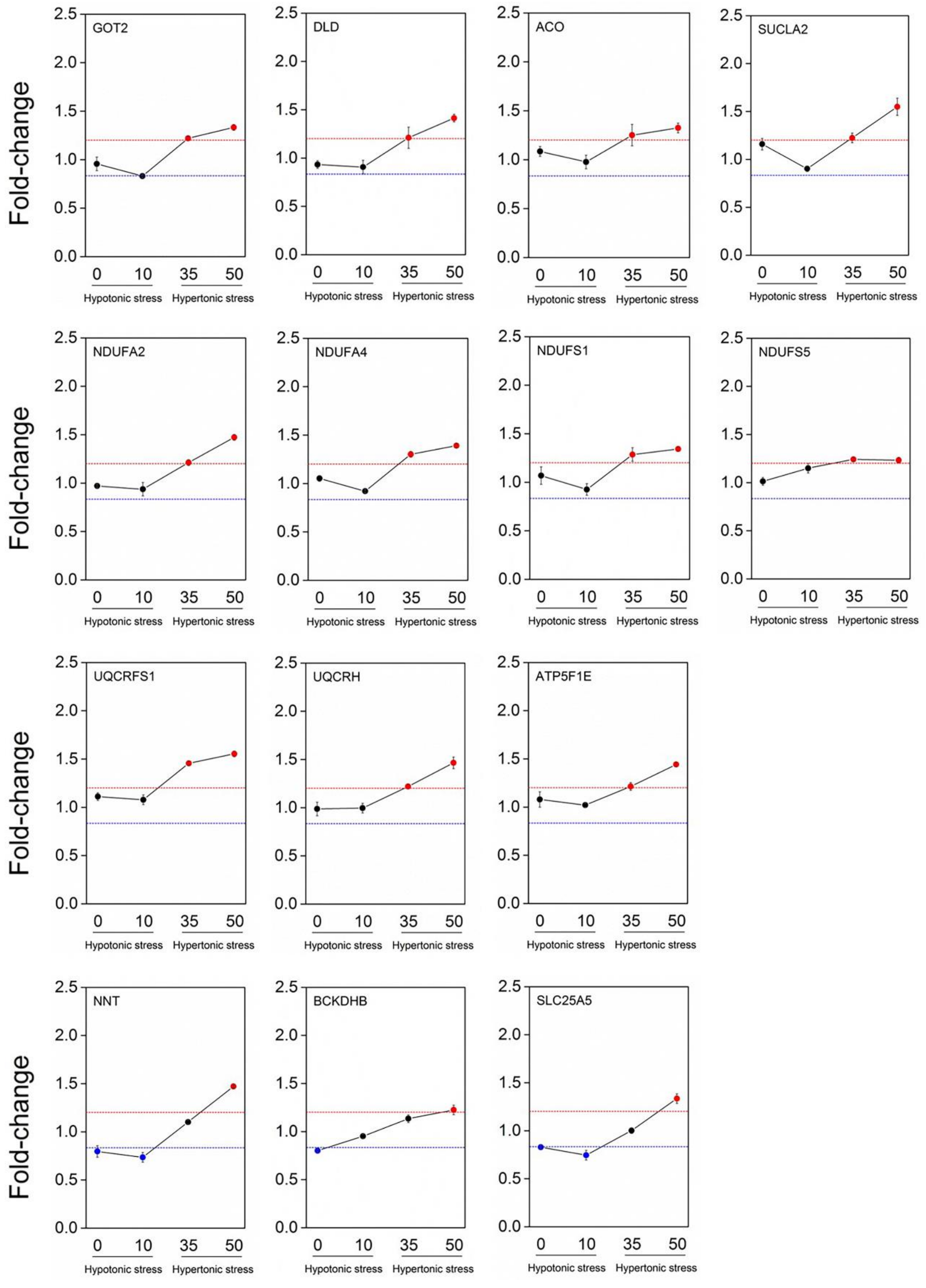

Figure S4. Expression levels of 14 DEPs related to energy metabolism in the gills of *S. argus* exposed to different salinity environments identified by iTRAQ technology. The red dotted line represents a significant up-regulation with a threshold of 1.2-fold, and the blue dotted line represents

a significant down-regulation with a threshold of 0.83-fold. Blue circles represent fold change < 0.83, red circles represent fold change > 1.2, and black circles represent 0.83 < fold change < 1.2.
